# Glycomic Characterization of Respiratory Tract Tissues of Ferrets: IMPLICATIONS FOR ITS USE IN INFLUENZA VIRUS INFECTION STUDIES*[Fn FN2]

**DOI:** 10.1074/jbc.M114.588541

**Published:** 2014-08-18

**Authors:** Nan Jia, Wendy S. Barclay, Kim Roberts, Hui-Ling Yen, Renee W. Y. Chan, Alfred K. Y. Lam, Gillian Air, J. S. Malik Peiris, Anne Dell, John M. Nicholls, Stuart M. Haslam

**Affiliations:** From the ‡ Department of Life Sciences, Imperial College London, South Kensington Campus, London SW7 2AZ, United Kingdom,; the § Faculty of Medicine, Division of Infectious Disease, Imperial College London, Norfolk Place, London W2 1PG, United Kingdom,; the ¶ School of Public Health, University of Hong Kong, Pokfulam, Hong Kong SAR, China,; the ‖ Department of Pathology, Griffith University, 4111 Queensland, Australia,; the ** Department of Biochemistry and Molecular Biology, University of Oklahoma Health Sciences Center, Oklahoma City, Oklahoma 73126-0901, and; the ‡‡ Department of Pathology, University of Hong Kong, Pokfulam, Hong Kong SAR, China

**Keywords:** Carbohydrate Structure, Glycomics, Influenza Virus, Mass Spectrometry (MS), Sialic Acid

## Abstract

The initial recognition between influenza virus and the host cell is mediated by interactions between the viral surface protein hemagglutinin and sialic acid-terminated glycoconjugates on the host cell surface. The sialic acid residues can be linked to the adjacent monosaccharide by α2–3- or α2–6-type glycosidic bonds. It is this linkage difference that primarily defines the species barrier of the influenza virus infection with α2–3 binding being associated with avian influenza viruses and α2–6 binding being associated with human strains. The ferret has been extensively used as an animal model to study the transmission of influenza. To better understand the validity of this model system, we undertook glycomic characterization of respiratory tissues of ferret, which allows a comparison of potential viral receptors to be made between humans and ferrets. To complement the structural analysis, lectin staining experiments were performed to characterize the regional distributions of glycans along the respiratory tract of ferrets. Finally, the binding between the glycans identified and the hemagglutinins of different strains of influenza viruses was assessed by glycan array experiments. Our data indicated that the respiratory tissues of ferret heterogeneously express both α2–3- and α2–6-linked sialic acids. However, the respiratory tissues of ferret also expressed the Sda epitope (NeuAcα2-3(GalNAcβ1–4)Galβ1–4GlcNAc) and sialylated *N,N*′-diacetyllactosamine (NeuAcα2–6GalNAcβ1–4GlcNAc), which have not been observed in the human respiratory tract surface epithelium. The presence of the Sda epitope reduces potential binding sites for avian viruses and thus may have implications for the usefulness of the ferret in the study of influenza virus infection.

## Teaser

**Background:** The ferret is a key animal model to study the transmission characteristics of influenza viruses.

**Results:** Characterization of ferret respiratory tract tissues identified influenza virus glycan receptors.

**Conclusion:** Species-specific influenza virus glycan receptors were identified.

**Significance:** Our findings provide new insights into the usefulness of ferrets in the study of influenza virus infection.

## Introduction

Aquatic birds are the natural hosts of the different subtypes of influenza viruses, apart from two newly isolated strains in bats (1). The ability of the virus to transmit to multiple species is dependent on many host and viral factors, one of the first being the interaction of the viral hemagglutinin (HA) with host sialic acid (Sia)^4^ ligands. Previous studies have indicated that host determination may be dependent on the linkage of the Sia with its adjacent galactose. Thus α2–6-linked Sia preferentially binds human adapted viruses, and α2–3-linked Sia preferentially binds avian-derived viruses. As an example of this restriction, previous studies suggested that avian viruses, especially highly pathogenic avian influenza of the H5 and H7 subtypes, should not be able to infect the human airway epithelium. Previous lectin binding studies indicated that human airway epithelium contained primarily α2–6-linked Sia. This would indicate a requirement for the virus to infect an intermediate host such as the pig, which was reported to contain both α2–3- and α2–6-linked Sia where it will gradually acquire the ability to bind to α2–6-Sia through mutations. The 1997 H5N1 outbreak, however, indicated that this paradigm was too simple as this highly pathogenic avian influenza (which showed a restricted α2–3-Sia binding) was able to directly infect human airways, leading to severe pulmonary disease, but was limited to human to human transmission.

In an attempt to understand the mechanism of the severe disease caused by influenza, the ferret has been used as a model to explore the pathophysiology of the virus and host interaction. The ferret appears to be an excellent model to study influenza infection and transmission for two main reasons: ferrets are naturally susceptible to influenza infection without prior host adaptation, and disease severity appears to mimic that of natural infection of humans (2). The ferret has been used as a model since the 1970s (Belser *et al.* (3)), when Potter *et al.* (4) found that adult ferrets could be infected with egg-passaged H3N2 viruses with nasal washings all positive. A difference in replication between the trachea and nasal mucosa was shown by Basarab and Smith (5), where tracheal production of H2N2 was 0.001% of nasal mucosal infection. These early studies therefore documented that virus replication occurs predominantly in the ferret nasal mucosa with much less in the trachea.

The limitations of the ferret model to predict disease severity in humans was tested in the 2009 pandemic. Ferret studies published during the early stages of the outbreak indicated that disease severity was greater than that seen in seasonal H1N1 infection (6) and approached that seen with H5N1 infection (7), yet human data eventually indicated that the disease severity was not as great as initially suggested (8). Furthermore, in the recent H7N9 outbreak, although *ex vivo* studies showed replication in the bronchus (9), Richard *et al.* (10) did not find evidence of replication of viral antigen in the trachea using A/Anhui/1/2013, but antigen was detected in the nasal respiratory and olfactory epithelium. In contrast, Xu *et al.* (11), also using A/Anhui/1/2013, detected virus in the trachea and olfactory bulb and postulated that the difference in findings may be due to different populations of ferrets used. Belser *et al.* (12) also found elevated viral titers in the nasal turbinates and trachea using both A/Anhui/1/2013 and A/Shanghai/1/2013. In that study, glycan array analysis showed low binding of Shanghai/1 to α2–6-sialylated glycans but high binding of A/Anhui/1/2013 to α2–6-sialylated glycans. Kreijtz *et al.* (13) used 12-month-old ferrets and found several tracheal cells were positive for nucleoprotein without evidence of inflammation. The bronchi, however, showed epithelial necrosis and submucosa glandular adenosis. All animals had an acute necrotizing (bronco) interstitial pneumonia.

Because one of the determinants of infection is affinity of the virus for sialylated glycans, it is essential for there to be an understanding of the types of glycans present in the ferret respiratory tract. The first study to investigate the differential expression of α2–3- *versus* α2–6-linked Sia was done by Leigh *et al.* in 1995 (14). Using fluorescently labeled lectins, SNA (which binds α2–6-sialylated glycans) bound to the cilia of tracheal airways, but MAA (which binds to α2–3-sialylated glycans) only bound to the submucosa and not to the epithelium of weanling 7–8-week-old ferrets. This study has been cited by subsequent authors as supporting the usefulness of ferrets for influenza studies, because the high expression of α2–6-Sia in the conducting airways and the presence of α2–3-Sia in the lung parenchyma has been suggested to be representative of the human system.

However, there are potential issues with lectin binding histochemistry because the results may depend on the lectin supplier and methodology used. Furthermore, lectin binding only identifies the nature of the Sia and adjacent galactose, but research by Gambaryan *et al.* (15) showed that other subterminal sugars play an important role in the recognition of the binding of influenza viruses to the receptor. Therefore, there exists a need to determine what actual glycans are present in the respiratory tract of different species susceptible to influenza viruses. The most effective technique to characterize the complex mixtures of glycans that are derived from mammalian cells and tissues is mass spectrometry via the application of integrated glycomic workflows (16, 17). We have developed and applied such workflows to characterize respiratory tract Sia glycan receptors from a range of species. The first of these studies was done on swine respiratory epithelial cells by Bateman *et al.* (18), followed by human respiratory tract studies by Walter *et al.* (19), swine respiratory tract by van Poucke *et al.* (20), and then mouse lung by Bern *et al.* (21). Although mice offer many advantages as an animal model, including low cost and the availability of transgenic mice, their lack of efficient influenza viral replication and clinical signs have made them poor systems to study the transmission of influenza viruses (22). Moreover, the respiratory tissues of swine and mice have been shown to express significant levels of Galα1–3Gal and *N*-glycolylneuraminic acid (NeuGc)-terminated glycans, which are not produced by humans (23, 24). To understand how the glycosylation of ferret respiratory tissues differ from other animal models and humans, we demonstrate a comprehensive analysis of ferret upper and lower respiratory tract glycosylation.

## EXPERIMENTAL PROCEDURES

### Ferret Respiratory Tract Tissues for Histochemistry

Male and female ferrets were used with an age range of 6 months to 3 years. Ferret tissues from the United States (Triple F Farms) were from 6-month-old ferrets, as used in previous publications (25, 26). Two ferrets from Hong Kong were imported from New Zealand and were used at an age of over 36 months.

### Glycomic Analysis

Ferrets for glycomic experiments were seronegative and 16–24 weeks old (27). All animal research described in this study was carried out under a United Kingdom Home Office License PPL/70/6643. Female ferrets were anesthetized by intramuscular injection of ketamine (25 mg kg^−1^) and xylazine (1.5 mg kg^−1^), followed by intraperitoneal injection of a lethal overdose of sodium pentobarbital. Extracted tissues were kept on ice until further processing. Ferrets were nasal washed, while conscious, by instilling 2 ml of PBS in five separate 400-ml volumes into the nostrils while the animals were held in a prone position. Animals were then brought upright again, and the expelled fluid was collected in modified 250-ml centrifuge tubes. Nasal wash samples were kept on ice until further processing. Five ferret tissues, lung, trachea, soft palate, nasal turbinate, and nasal wash, were analyzed. Detailed sample preparation protocol for mass spectrometric (MS) analysis has been described previously (19). Briefly, the lungs and tracheas were separately homogenized and sonicated in ice-cold ultra-pure water. Glycolipids were extracted by chloroform/methanol/water, followed by the release of lipid-linked glycans via rEGCase II (Takara) digestion and a subsequent deuteroreduction step. Glycoproteins were reduced, carboxymethylated, and tryptic digested prior to the release of protein-linked *N*-glycans by PNGase F (Roche Applied Science) digest and *O-*linked glycans by reductive elimination. Soft palate and nasal turbinate tissues were suspended in lysis buffer (25 mm Tris, 150 mm NaCl, 5 mm EDTA, and 1% CHAPS (v/v), pH 7.4) before homogenization and sonication were performed. The homogenates were subsequently dialyzed against a 50 mm ammonia bicarbonate buffer, after which the samples were lyophilized and processed as above. The ferret nasal wash was dialyzed directly against a 50 mm ammonia bicarbonate buffer, lyophilized, and processed as above. All released *N*-, *O-,* and lipid-derived glycans were permethylated prior to MS analysis.

Purified, un-derivatized glycans were incubated with sialidase A (ProZyme, GK80040), sialidase S (ProZyme, GK80020), or endo-β-galactosidase (Seikagaku) separately. The digested samples were lyophilized and permethylated prior to MS analysis.

### Mass Spectrometric Analysis

Glycomic profiles were acquired on a matrix-assisted laser desorption ionization-time of flight (MALDI-TOF) mass spectrometer from Voyager-DE^TM^ STR PerSeptive Biosystems. Permethylated samples were dissolved in 10 μl of methanol from which 0.5 μl was added to 0.5 μl of the matrix (20 mg/ml of 2,5-dihydrobenzoic acid in 70% (v/v) aqueous methanol). The sample/matrix mixture was then loaded onto the sample plate for the mass spectrometric analysis. MALDI-TOF/TOF experiments were performed with a 4800 Proteomics Analyzer (Applied Biosystems) using 3,4-diaminobenzophenone (20 mg/ml in 70% (v/v) aqueous acetonitrile) as the matrix. Both instruments were operated in the reflectron positive mode. All MS and MS/MS data were processed using Data Explorer 4.9 software (Applied Biosystems).

### GC/MS Linkage Analysis

GC-MS linkage analysis of partially methylated alditol acetates was carried out on a PerkinElmer Life Sciences Clarus 500 instrument fitted with RTX-5 fused capillary column (30 m × 0.32 mm internal diameter, Restec Corp.). Partially methylated alditol acetates were prepared from permethylated samples as described previously (17). The permethylated glycans were hydrolyzed with 2 m trifluoroacetic acid for 2 h at 121 °C, reduced with 10 mg/ml sodium borodeuteride in 2 m aqueous ammonium hydroxide at room temperature, and acetylated with acetic anhydride at 100 °C for 1 h. The sample was dissolved in hexanes and injected onto the column at 60 °C. The column was maintained at this temperature for 1 min and then heated to 300 °C at a rate of 8 °C/min.

### Details of Experimental Data Utilized to Define Glycan Structures

#### 

##### Ferret Lung N-Glycans

1) All *N*-glycans were assumed to have a core of Manα1–6(Manα1–3)Manβ1–4GlcNAcβ1-4GlcNAc based on known biosynthetic pathways and susceptibility to PNGase F digestion. 2) Monosaccharide compositions in terms of numbers of Hex, HexNAc, etc. derived from MALDI-MS in the +ve ion mode of molecular ions of PNGase F-released permethylated species were assigned manually. 3) MALDI-TOF/TOF MS/MS fragmentation was from the following molecular ions: *m*/*z* 2431, 2809, 3007, 3211, 3252, 3456, and 3906. Fragment ions were identified manually and with the assistance of the Glycoworkbench tool (Version 1.2). 4) GC-MS linkage analysis was from partially methylated alditol acetates. 5) Sialidase A digestion followed by monosaccharide compositions in terms of numbers of Hex, HexNAc, etc. were derived from MALDI-MS in the +ve ion mode of molecular ions of PNGase F-released permethylated species, which were assigned manually. 6) Sialidase A digestion was followed by MALDI-TOF/TOF MS/MS fragmentation of the following molecular ions: *m*/*z* 3183, 3591, 4531, and 4775. Fragment ions were identified manually and with the assistance of the Glycoworkbench tool (Version 1.2). 7) Sialidase S digestion was followed by monosaccharide compositions in terms of numbers of Hex, HexNAc, etc. derived from MALDI-MS in the +ve ion mode of molecular ions of PNGase F-released permethylated species, which were assigned manually. 8) Sialidase S digestion was followed by MALDI-TOF/TOF MS/MS fragmentation of the following molecular ions: *m*/*z* 3211, 3456, 3905, 4267, 4716, and 4852. Fragment ions were identified manually and with the assistance of the Glycoworkbench tool (Version 1.2). 9) Endo-β-galactosidase digestion was followed by monosaccharide compositions in terms of numbers of Hex, HexNAc, etc. derived from MALDI-MS in the +ve ion mode of molecular ions of PNGase F-released permethylated species, which were assigned manually. 10) Endo-β-galactosidase digestion was followed by MALDI-TOF/TOF MS/MS fragmentation of the following molecular ions: *m*/*z* 879, 1083, 1124 and 1328. Fragment ions were identified manually and with the assistance of the Glycoworkbench tool (Version 1.2).

##### Ferret Lung O-Glycans

1) All *O-*glycans were assumed to have reducing end GalNAc based on known biosynthetic pathways. 2) Monosaccharide compositions in terms of numbers of Hex, HexNAc, etc. derived from MALDI-MS in the +ve ion mode of molecular ions of reductively eliminated permethylated species were assigned manually. 3) MALDI-TOF/TOF MS/MS fragmentations are of the following molecular ions: *m*/*z* 895, 1140, and 1501. Fragment ions were identified manually and with the assistance of the Glycoworkbench tool (Version 1.2). 4) Sialidase A digestion followed by monosaccharide compositions in terms of numbers of Hex, HexNAc, etc. derived from MALDI-MS in the +ve ion mode of molecular ions of reductively eliminated permethylated species were assigned manually. 5) Sialidase A digestion was followed by MALDI-TOF/TOF MS/MS fragmentation of the following molecular ions: *m*/*z* 779, 983, and 1502. Fragment ions were identified manually and with the assistance of the Glycoworkbench tool (Version 1.2). 6) Sialidase S digestion followed by monosaccharide compositions in terms of numbers of Hex, HexNAc, etc. derived from MALDI-MS in the +ve ion mode of molecular ions of reductively eliminated permethylated species were assigned manually. 7) Sialidase S digestion was followed by MALDI-TOF/TOF MS/MS fragmentation of the following molecular ions: *m*/*z* 895 and 1140. Fragment ions were identified manually and with the assistance of the Glycoworkbench tool (Version 1.2).

##### Ferret Lung Glycolipid-derived Glycans

1) All glycolipid-derived glycans were assumed to have a core of Galβ1–4Glc based on known biosynthetic pathways and susceptibility to rEGCase II digestion. 2) Monosaccharide compositions in terms of numbers of Hex, HexNAc, etc. derived from MALDI-MS in the +ve ion mode of molecular ions of rEGCase II-released permethylated species were assigned manually. 3) MALDI-TOF/TOF MS/MS fragmentation of the following molecular ions: *m*/*z* 855, 1216, 1304, 1549, 1753, 1841, 1998, and 2203. Fragment ions were identified manually and with the assistance of the Glycoworkbench tool (Version 1.2). 4) Sialidase A digestion followed by monosaccharide compositions in terms of numbers of Hex, HexNAc, etc. derived from MALDI-MS in the +ve ion mode of molecular ions of rEGCase II-released permethylated species were assigned manually. 5) Sialidase A digestion was followed by MALDI-TOF/TOF MS/MS fragmentation of the following molecular ions: *m*/*z* 943, 1147, 1188, 1637, and 1841. Fragment ions were identified manually and with the assistance of the Glycoworkbench tool (Version 1.2). 6) Sialidase S digestion followed by monosaccharide compositions in terms of numbers of Hex, HexNAc, etc. derived from MALDI-MS in the +ve ion mode of molecular ions of rEGCase II-released permethylated species were assigned manually. 7) Sialidase S digestion was followed by MALDI-TOF/TOF MS/MS fragmentation of the following molecular ions: *m*/*z* 943, 1216, 1304, 1549, 1753, 1998, and 2203. Fragment ions were identified manually and with the assistance of the Glycoworkbench tool (Version 1.2).

##### Ferret Trachea N-Glycans

1) All *N*-glycans were assumed to have a core of Manα1–6(Manα1–3)Manβ1–4GlcNAcβ1-4GlcNAc based on known biosynthetic pathways and susceptibility to PNGase F digestion. 2) Monosaccharide compositions in terms of numbers of Hex, HexNAc, etc. derived from MALDI-MS in the +ve ion mode of molecular ions of PNGase F-released permethylated species were assigned manually. 3) MALDI-TOF/TOF MS/MS fragmentation of the following molecular ions is shown: *m*/*z* 3211, 3660, 3719, 3835, 3893, 3906, 4168, 4342, and 4587. Fragment ions were identified manually and with the assistance of the Glycoworkbench tool (Version 1.2).

##### Ferret Trachea O-Glycans

1) All *O-*glycans were assumed to have reducing end GalNAc based on known biosynthetic pathways. 2) Monosaccharide compositions in terms of numbers of Hex, HexNAc, etc. derived from MALDI-MS in the +ve ion mode of molecular ions of reductively eliminated permethylated species were assigned manually.

##### Ferret Trachea Glycolipid-derived Glycans

1) All glycolipid-derived glycans were assumed to have a core of Galβ1–4Glc based on known biosynthetic pathways and susceptibility to rEGCase II digestion. 2) Monosaccharide compositions in terms of numbers of Hex, HexNAc, etc. derived from MALDI-MS in the +ve ion mode of molecular ions of rEGCase II-released permethylated species were assigned manually. 3) MALDI-TOF/TOF MS/MS fragmentation of the following molecular ions is shown: *m*/*z* 1216, 1305, 1551, 1754, and 2203. Fragment ions were identified manually and with the assistance of the Glycoworkbench tool (Version 1.2).

##### Ferret Soft Palate N-Glycans

1) All *N*-glycans were assumed to have a core of Manα1–6(Manα1–3)Manβ1–4GlcNAcβ1-4GlcNAc based on known biosynthetic pathways and susceptibility to PNGase F digestion. 2) Monosaccharide compositions in terms of numbers of Hex, HexNAc, etc. derived from MALDI-MS in the +ve ion mode of molecular ions of PNGase F-released permethylated species were assigned manually. 3) MALDI-TOF/TOF MS/MS fragmentation of the following molecular ion is shown: *m*/*z* 2448, 2809, 3024, 3211, 3835, 3893, and 4022. Fragment ions were identified manually and with the assistance of the Glycoworkbench tool (Version 1.2).

##### Ferret Soft Palate O-Glycans

1) All *O-*glycans were assumed to have reducing end GalNAc based on known biosynthetic pathways. 2) Monosaccharide compositions in terms of numbers of Hex, HexNAc, etc. derived from MALDI-MS in the +ve ion mode of molecular ions of reductively eliminated permethylated species were assigned manually.

##### Ferret Nasal Turbinate N-Glycans

1) All *N*-glycans were assumed to have a core of Manα1–6(Manα1–3)Manβ1-4GlcNAcβ1–4GlcNAc based on known biosynthetic pathways and susceptibility to PNGase F digestion. 2) Monosaccharide compositions in terms of numbers of Hex, HexNAc, etc. derived from MALDI-MS in the +ve ion mode of molecular ions of PNGase F-released permethylated species were assigned manually. 3) MALDI-TOF/TOF MS/MS fragmentation of the following molecular ions is shown: *m*/*z* 2431, 2809, 3037, 3054, 3211, 3299, 3415, 3661, and 4022. Fragment ions were identified manually and with the assistance of the Glycoworkbench tool (Version 1.2).

##### Ferret Nasal Wash N-Glycans

1) All *N*-glycans were assumed to have a core of Manα1–6(Manα1–3)Manβ1-4GlcNAcβ1–4GlcNAc based on known biosynthetic pathways and susceptibility to PNGase F digestion. 2) Monosaccharide compositions in terms of numbers of Hex, HexNAc, etc. derived from MALDI-MS in the +ve ion mode of molecular ions of PNGase F-released permethylated species were assigned manually. 2) MALDI-TOF/TOF MS/MS fragmentation of the following molecular ions is shown: *m*/*z* 2850, 3007, 3211, 4021, and 4268. Fragment ions were identified manually and with the assistance of the Glycoworkbench tool (Version 1.2).

##### Ferret Nasal Wash O-Glycans

1) All *O-*glycans were assumed to have reducing end GalNAc based on known biosynthetic pathways. 2) Monosaccharide compositions in terms of numbers of Hex, HexNAc, etc. derived from MALDI-MS in the +ve ion mode of molecular ions of reductively eliminated permethylated species were assigned manually. Details provided were guided by MIRAGE (28)

### Lectin Binding

Human bronchus and lung samples were obtained from patients undergoing resection for lung tumors and archived formalin-fixed paraffin-embedded material was used. The collection of human respiratory tract tissues was approved by the University of Hong Kong/Hospital Authority Hong Kong West Cluster (HKU/HA HKW IRB) with written informed consent provided by study participants and/or their legal guardians. We used lung tissues from Caucasian (*n* = 10) and Asian (*n* = 20) patients. Tissue samples were obtained from formalin-fixed and paraffin-embedded tissues and stained for DBA lectin histochemistry and CT1 immunohistochemistry using methods below.

We used biotinylated SNA, MAA-I, and MAA-II from Vector Laboratories, and a biotinylated DBA from Vector Laboratories (DBA-I). Formalin-fixed and paraffin-embedded tissues were sampled, and slides were incubated with the above lectins at previously documented concentrations. Cad/Sda was detected using a monoclonal antibody provided by K. Klisch (University of Nottingham). Double staining using DBA and influenza nucleoprotein was used for ferret tissues that had been infected with pandemic H1N1 viruses and then euthanized (26).

### Enzyme Expression

Monoclonal or polyclonal antibodies to the following sialyltransferases were purchased from the following companies with antibodies used at recommended concentrations: β4GALT1 (Sigma) 1:100; β4GALNT2 (Abnova) 1:200; ST3GAL3 (Novus) 1:200; ST3GAL4 (Novus) 1:100; ST3GAL6 (Novus) 1:200; ST6GAL1 (R&D) 1:50; ST6GALNAC1 (Novus) 1:500, and ST6GALNAC2 (Novus) 1:1000. If antigen retrieval resulted in a clear background, citrate buffer was utilized. Detection was with either alkaline phosphatase or Vector Red as detailed previously.

### Glycan Binding

We used glycan binding data from previously studied and published viruses. Binding to sialylated glycans on the Consortium for Functional Glycomics Version 5.1 array was analyzed, and the effects of a terminal GalNAc on binding was evaluated using the glycans GalNAcβ1–4(Neu5Acα2–3)Galβ1–4GlcNAcβ-Sp8,GalNAcβ1–4(Neu5Acα2–3)Galβ1-4Glcβ-Sp0, Neu5Acα2–3Galβ1–4GlcNAcβ-Sp8, and Neu5Acα2-3Galβ1–4Glcβ-Sp0.

### Lectin Binding to Glycan Array

Commercially available lectins MAA-I, MAA-II, SNA (Vector Laboratories), SNA I (EY Labs), and biotinylated DBA I (Vector Laboratories) were used to bind to Version 5.1 of the array, and results were plotted as a relative binding intensity.

## RESULTS

### 

#### 

##### Ferret Lung Glycoproteins

**FIGURE 1.**
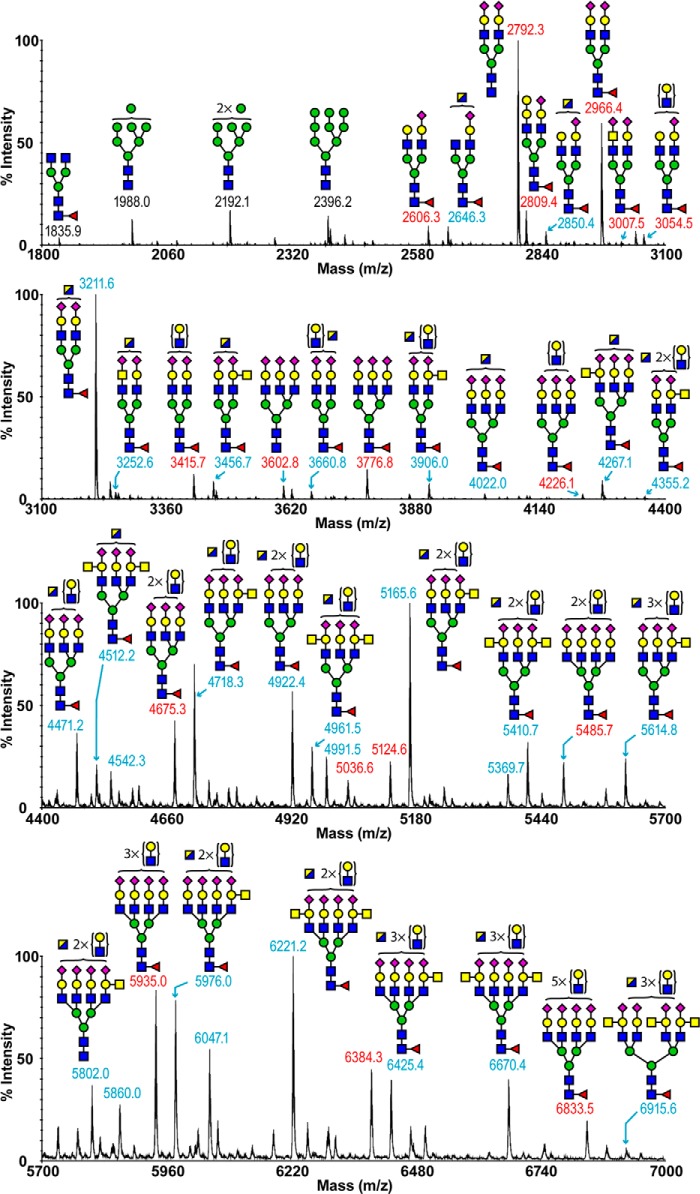
***N*-Glycan profile of ferret lung.** The MALDI-TOF spectra of permethylated *N*-glycans of ferret lung were obtained from the 50% acetonitrile fraction from a C18 Sep-Pak column. All molecular ions detected are present in the form of [M + Na]^+^. Annotations include major structures that are unsialylated (*black*) and sialylated (*red*) and glycans carrying the Sda epitope (*blue*). Putative structures are based on composition, tandem MS, and biosynthetic knowledge. See “Experimental Procedures” for full details of experiments that provided data for structural assignments. Structures that show sugars outside of a *bracket* have not been unequivocally defined. A full list of structural compositions is available in supplemental Table S1. Color symbols are as follows: *yellow square*, GalNAc; *blue square*, GlcNAc; *half-blue/half-yellow square*, GalNAc or GlcNAc; *yellow circle*, galactose; *green circle*, mannose; *purple diamond*, *N*-acetylneuraminic acid; *red triangle*, fucose.

The MALDI-TOF spectrum of ferret lung *N*-linked glycans revealed diverse structural variations up to *m*/*z* 7000 (Fig. 1, supplemental Table S1). The low mass region of the spectrum was dominated by high mannose structures (*m*/*z* 1988, 2192, and 2396), whereas middle and high *m*/*z* regions consisted of molecular ions that were consistent with bi-, tri-, and tetra-antennary complex glycans, including structures with LacNAc repeats. The majority of complex glycans were mono-fucosylated, suggesting core fucosylation. The major nonreducing terminal modification was the sialylation of LacNAc with NeuAc. Sialylation with NeuGc was not detected. Of particular note was a series of abundant complex glycans (*m*/*z* 2850–6915), whose compositions were consistent with the presence of the Sda epitope (NeuAcα2–3(GalNAcβ1–4) Galβ1–4GlcNAc) on some of their antennae. Additional terminal structures included sialylated LacdiNAc (NeuAc-GalNAcβ1–4GlcNAc, *m*/*z* 3007 and 3252) and Galα1–3Gal (*m*/*z* 2809).

To provide additional levels of structural definition, the majority of molecular ions were subjected to MALDI-TOF/TOF MS/MS analysis. Exemplar data are displayed in supplemental Fig. S1. A general feature was that all mono-fucosylated complex glycans gave a y-ion at *m*/*z* 474, arising from cleavage at the chitobiose core, which confirmed that the single fucose residue was located exclusively on the *N*-glycan core. Also, Galα1–3Gal-containing structures produced a strong b-ion at *m*/*z* 690. The fragmentation of the molecular ion at *m*/*z* 3252 (supplemental Fig. S1) produced a b-ion at *m*/*z* 1092, which is diagnostic of the tetrasaccharide constituent of the Sda capping group, although the y-ion at 2183 represented the loss of the Sda tetrasaccharide from the molecular ion. An additional structural isomer is also present, which lacks the Sda capping and has a bisecting GlcNAc instead of the terminal GalNAc of the Sda. Finally, the b-ion of *m*/*z* 888 was the signature ion for a sialylated LacdiNAc unit, and its complementary y-ion was observed at *m*/*z* 2387.

**FIGURE 2.**
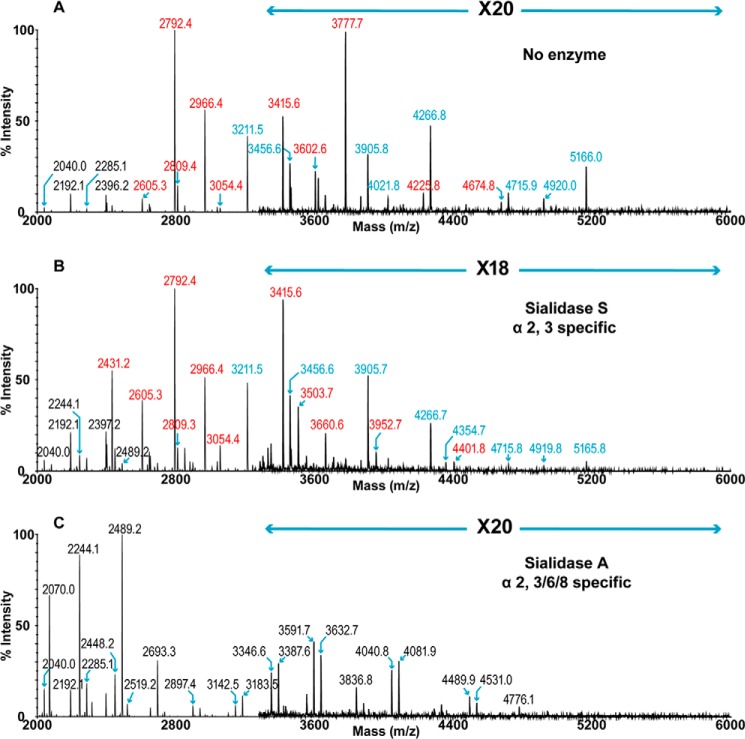
***N*-Glycan profiles of ferret lung following sialidase treatment.** *A,* MALDI-TOF spectrum of permethylated *N*-glycans. *B,* MALDI-TOF spectrum of permethylated *N*-glycans after digestion with sialidase S. *C,* MALDI-TOF spectrum of permethylated *N*-glycans after digestion with sialidase A. Annotations include major structures that are unsialylated (*black*), sialylated (*red*), and glycans carrying the Sda epitope (*blue*). All molecular ions detected are present in the form [M + Na]^+^. Color symbols are as follows: *yellow square*, GalNAc; *blue square*, GlcNAc; *half-blue/half-yellow square,* GalNAc or GlcNAc; *yellow circle*, galactose; *green circle*, mannose; *purple diamond*, N-acetylneuraminic acid; *red triangle*, fucose.

Sia typically links to its adjacent monosaccharide via α2–3 or α2–6 configuration, and it is this linkage difference that primarily defines the species barrier of the influenza virus. Therefore, the linkage status of Sia was assessed via digestion with linkage-specific enzymes. Sialidase A removes both α2–3- and α2–6-linked Sia, although sialidase S specifically releases Sia that is in α2–3-linkage. Compared with untreated lung (Fig. 2*A*), treatment with sialidase A resulted in a complete desialylation of ferret lung *N*-glycans (Fig. 2*C*). Bi-antennary glycans were the major reaction products (*m*/*z* 2070, 2244, and 2489), which were consistent with the dominant presence of their corresponding sialylated structures prior to the digestion (*m*/*z* 2792, 2966, and 3211). High mass range glycans bearing multiple LacNAc units (*e.g. m*/*z* 2692, 3591, 4040, and 4776) were observed, which again confirmed that the ferret lung contained tri- and tetra-antennary complex glycans with variable lengths of LacNAc extension. Additionally, a series of glycans whose compositions were consistent with terminal GalNAc (*e.g. m*/*z* 3183, 3632, 4081, 4531, and 4776), which represented the cleavage products of Sda structures. The MS spectrum of sialidase S-treated glycans revealed a partial desialylation, indicating the presence of Sia in both α2–3- and α2–6-linkage, with the latter being more abundant (Fig. 2*B*). The bi-antennary, di-sialylated glycans (*m*/*z* 2792 and 2966) remained the major compositions after the enzymatic digestion. However, the increase in relative intensities of mono-sialylated, bi-antennary structures (*m*/*z* 2431, 2605, and 3054) suggested that the Sia presented by each antenna of the same bi-antennary species may be a mixture of both α2–3- and α2–6-linkages. A striking feature of the spectrum is the incomplete digestion of the Sda-containing structures, even though Sda by definition is α2–3-sialylated (*e.g. m*/*z* 3211, 3905, and 4266). The resistance to different neuraminidase treatments has been reported previously (29, 30), and the neuraminidase from a H2N2 strain showed reduced activity toward Tamm-Horsfall glycoprotein, which is known to express Sda-containing structures (31, 32).

Endo-β-galactosidase was used to cleave poly-LacNAc extensions on the *N*-glycans. Digested fragments were produced at *m*/*z* 518 and 722 (supplemental Fig. S2), which provided solid evidence for the presence of poly-LacNAc extensions. The major nonreducing end capping groups were the sialylated LacNAc and Sda epitope (*m*/*z* 1083 and 1328, respectively), which is consistent with their frequent appearance in the MS spectrum of *N*-glycans (Fig. 1). Minor species of Galα1–3Gal were also observed (*m*/*z* 926), but no significant NeuGc was identified.

The total composition of the ferret lung derived *N*-linked glycans was also investigated by GC-MS linkage analysis (supplemental Table S2). The presence of 2-, 2,4-, and 2,6-linked mannose indicated the presence of bi-, tri-, and tetra-antennary complex glycans. Bisected complex glycans were also detected as evidenced by the presence of 3,4,6-linked mannose. The expression of the Sda epitope was confirmed by the presence of terminal GalNAc together with 3,4-linked Gal. The high levels of terminal Gal and 3-linked Gal are consistent with Galα1–3Gal capping. The 6-linked Gal peak is produced from the α2–6-sialylated structures, whereas the 3-linked Gal peak corresponds to the α2–3-sialylated glycans as well as Galα-Gal-terminated structures and poly-LacNAc extensions. By comparing the relative intensities between the two peaks, we were able to confirm the dominant expression of α2–6-sialylated glycans as is consistent with the sialidase digestions (Fig. 2). The presence of terminal fucose and 4,6-linked GlcNAc indicated core fucosylation. The 6-linked GalNAc confirms the α2–6-sialylation of LacdiNAc. The high abundance of 4-linked GlcNAc together with the lack of detection of any 3-linked GlcNAc indicates type 2 LacNAc units. This is also consistent with MS/MS fragmentation of multiple molecular ions.

**FIGURE 3.**
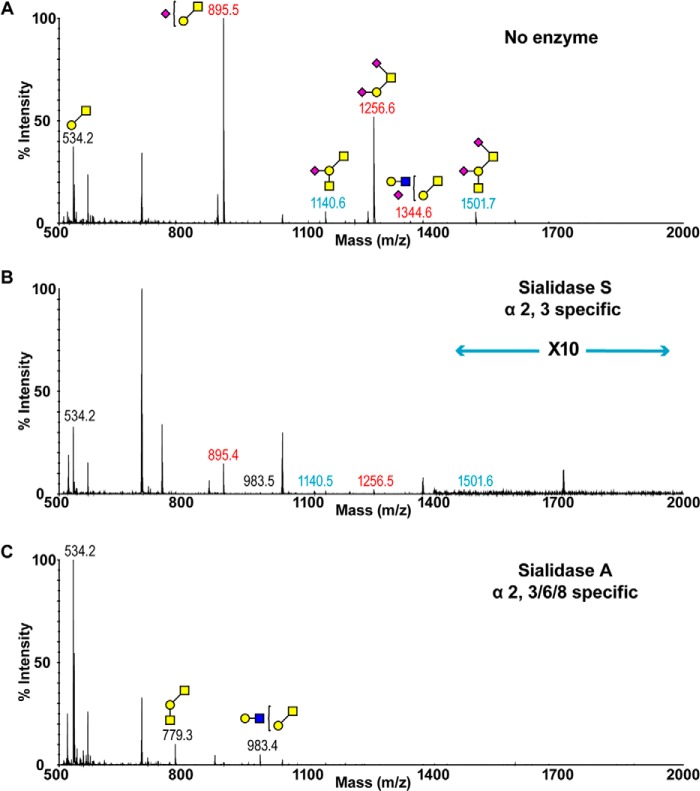
***O-*Glycan profiles of ferret lung before and after sialidase treatment.** *A,* MALDI-TOF spectrum of permethylated *O-*glycans. *B,* MALDI-TOF spectrum of permethylated *O-*glycans after digestion with sialidase S. *C,* MALDI-TOF spectrum of permethylated *O-*glycans after digestion with sialidase A. Annotations include major structures that are unsialylated (*black*), sialylated (*red*), and glycans carrying the Sda epitope (*blue*). All molecular ions detected are present in the form [M + Na]^+^. Putative structures are based on composition, tandem MS, and biosynthetic knowledge. See “Experimental Procedures” for full details of experiments that provided data for structural assignments. Structures that show sugars outside of a *bracket* have not been unequivocally defined. Color symbols are as follows: *yellow square*, GalNAc; *blue square*, GlcNAc; *yellow circle*, galactose; *purple diamond*, *N*-acetylneuraminic acid.

The main feature of ferret lung *O*-glycome was the rich expression of sialylated glycans (Fig. 3*A* and supplemental Table S3). Mono- and di-sialylated core 1 glycans were the most abundant structures (*m*/*z* 895 and 1256), which can be further elaborated to Sda-containing species (*m*/*z* 1140 and 1501). Structures were confirmed by MS/MS analysis (supplemental Fig. S1). Sialidase A fully removed α2–3- and α2–6-linked Sia, leading to an increase in the relative intensities of *m*/*z* 534 (Fig. 3*C*). The heterogeneous expression of α2–3- and α2–6-sialylated glycans was revealed by the MS spectrum of *O-*glycans treated with sialidase S (Fig. 3*B*). Sialo-glycans remaining after the digestion were sialylated in α2–6-linkage on the core GalNAc (*m*/*z* 895 and 1256). Trace levels of the Sda-presenting structure at *m*/*z* 1140 again indicated the partial resistance of this structure to the enzymatic digestion.

##### Ferret Lung Glycolipids

**FIGURE 4.**
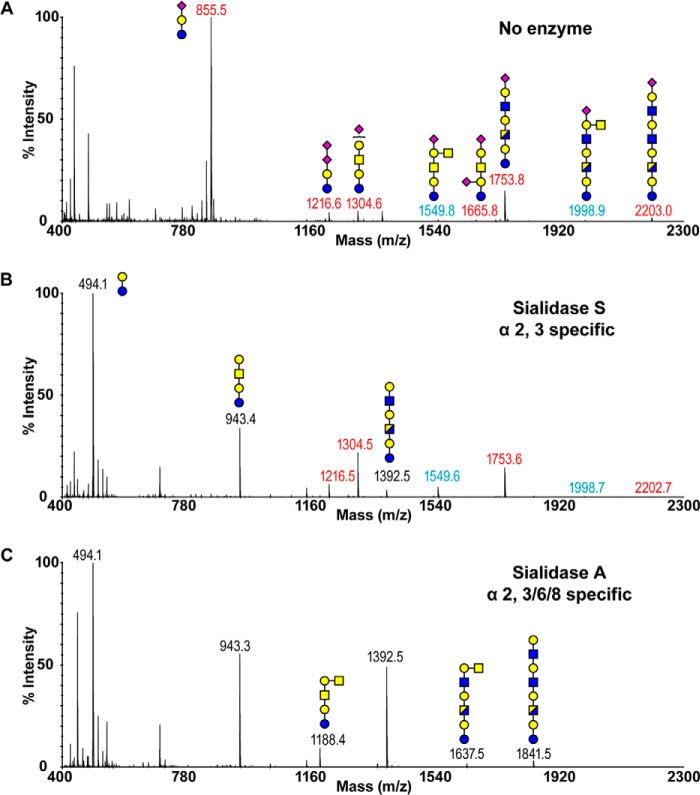
**Glycolipid-derived glycan profiles of ferret lung before and after sialidase treatment.** *A,* MALDI-TOF spectrum of permethylated, deuteroreduced glycolipids. *B,* MALDI-TOF spectrum of permethylated, deuteroreduced glycolipids after digestion with sialidase S. *C,* MALDI-TOF spectrum of permethylated, deuteroreduced glycolipids after digestion with sialidase A. Annotations include major structures that are unsialylated (*black*), sialylated (*red*), and glycans carrying the Sda epitope (*blue*). All molecular ions detected are present in the form [M + Na]^+^. Putative structures are based on composition, tandem MS, and biosynthetic knowledge. See “Experimental Procedures” for full details of experiments that provided data for structural assignments. Structures that show sugars outside of a *bracket* have not been unequivocally defined. Color symbols are as follows: *yellow square*, GalNAc; *blue square*, GlcNAc; *half-blue/half-yellow square*, GalNAc or GlcNAc; *yellow circle*, galactose; *purple diamond*, *N*-acetylneuraminic acid.

In addition to glycoproteins, glycolipids represent another major category of glycoconjugates expressed on the cell surface. The MALDI-TOF spectrum of ferret lung glycolipid-derived glycans (Fig. 4*A* and supplemental Table S4) clearly illustrated high levels of sialylation. A molecular ion consistent with the GM3 structure (*m*/*z* 855) dominated the spectrum with additional extended glycolipid-derived glycans identified. Although present in minor quantity, Sda epitopes were again observed (Fig. 4*A*, *m/z 1549* and *1998*), which was confirmed by characteristic fragmentation via MS/MS analysis (supplemental Fig. S1). Sialidase A entirely removed the Sia, resulting in a marked increase in the relative intensity of the desialylated lactose disaccharide (Fig. 4*C*, *m/z 494*). The peaks at *m*/*z* at 943 and 1392 correspond to structures with one or two additional HexHexNAc units on top of the lactose core, respectively (*m*/*z* 494). A peak at *m*/*z* 1188 was consistent with the composition of the desialylated Sda-containing structure (*m*/*z* 1549). The resistance of the Sda epitope to the hydrolysis by sialidase S was again demonstrated by the presence of structures at *m*/*z* 1549 and 1998 (Fig. 4*B*). The complete disappearance of GM3, forming the lactose core (*m*/*z* 494), illustrates the α2–3-linkage of the Sia. In contrast, the GD3 glycan (*m*/*z* 1216) remained prominent after the sialidase S treatment. This is expected because the outermost NeuAc is α2–8-linked (the Sia-Sia linkage was also indicated by MS/MS analysis of the *m*/*z* 1216 molecular ion). The heterogeneous expression of α2–3- and α2–6-linked NeuAc is revealed by the partial digestion of higher molecular weight species (*m*/*z* 1304, 1753, and 2202), forming their corresponding desialylated glycans (*m*/*z* 943, 1392, and 1841).

##### Ferret Trachea

**FIGURE 5.**
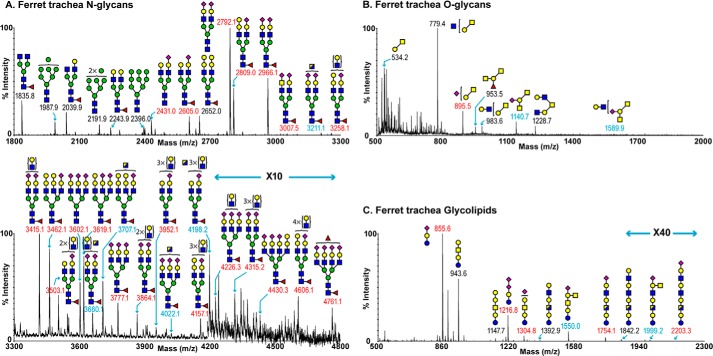
**Glycan profiles of ferret trachea.** *A,* MALDI-TOF spectrum of permethylated *N*-glycans. *B,* MALDI-TOF spectrum of permethylated *O-*glycans. *C,* MALDI-TOF spectrum of permethylated glycolipids. Putative structures are based on composition, tandem MS, and biosynthetic knowledge. Structures that show sugars outside of a *bracket* have not been unequivocally defined. See “Experimental Procedures” for full details of experiments that provided data for structural assignments. Annotations include major structures that are unsialylated (*black*), sialylated (*red*), and glycans carrying the Sda epitope (*blue*). All molecular ions detected are present in the form [M + Na]^+^. Color symbols are as follows: *yellow square*, GalNAc; *blue square*, GlcNAc; *half-blue/half-yellow square*, GalNAc or GlcNAc; *yellow circle,* galactose; *green circle*, mannose; *purple diamond*, *N*-acetylneuraminic acid; *red triangle*, fucose.

Our glycomic characterizations of ferret trachea included protein-derived *N*- and *O-*linked glycans as well as glycolipid-derived glycans (Fig. 5, *A–C*, and supplemental Tables S5–S7). The MALDI-TOF spectrum of ferret trachea-derived *N*-linked glycans revealed the presence of molecular ions that were consistent with high mannose structures (Fig. 5*A*, *m/z 1987, 2191,* and *2396*) and bi-, tri-, and tetra-antennary complex glycans displaying multiple LacNAc extensions up to *m*/*z* 4800. Similar to the ferret lung, molecular ions representing sialylated bi-antennary complex glycans (*m*/*z* 2792, 2809, and 2966) dominated the overall spectrum. The major nonreducing end modification was NeuAc-Gal-GlcNAc. Again, a series of complex glycans that have compositions consistent with the presence of the Sda epitope were prominent (*e.g. m*/*z* 3211, 3660, 3707, 4022, and 4198). In addition, two minor species expressing sialylated LacdiNAc were detected (*m*/*z* 3007 and 3252). A notable difference between the trachea *N*-glycans and the lung *N*-glycans was an increase in the relative abundance of species carrying Galα1–3Gal capping groups (*e.g. m*/*z* 2809, 3258, 3462, 3619, 4157, and 4430). As described previously, MS/MS analysis was performed on the majority of molecular ions and confirmed these structural features as well as core fucosylation.

The main distinguishing feature of the ferret trachea *O-*glycome is a relative increase in the expression of glycans consistent with core 2 structures (Fig. 5*B*, *m/z 779, 983, 1228,* and *1589*). Both core 1 and core 2 Sda-containing species were present (*m*/*z* 1140 and 1589).

The main feature of the glycolipids of the ferret trachea is a rich expression of sialylated structures (Fig. 5*C*). This was illustrated by the most abundant peak at *m*/*z* 855, representing the molecular ion of GM3. Additional sialo-glycans include GD3 (*m*/*z* 1216) and Sda-presenting structures (*m*/*z* 1550 and 1999) and multiple LacNAc units (*m*/*z* 1754, 2203).

##### Ferret Upper Respiratory Tract Tissues

To characterize the glycomic compositions of the ferret upper respiratory tract, we extracted structural information from soft palate, nasal turbinate, and ferret nasal wash, although the amount of starting material available for analysis was considerably less than for the lung.

**FIGURE 6.**
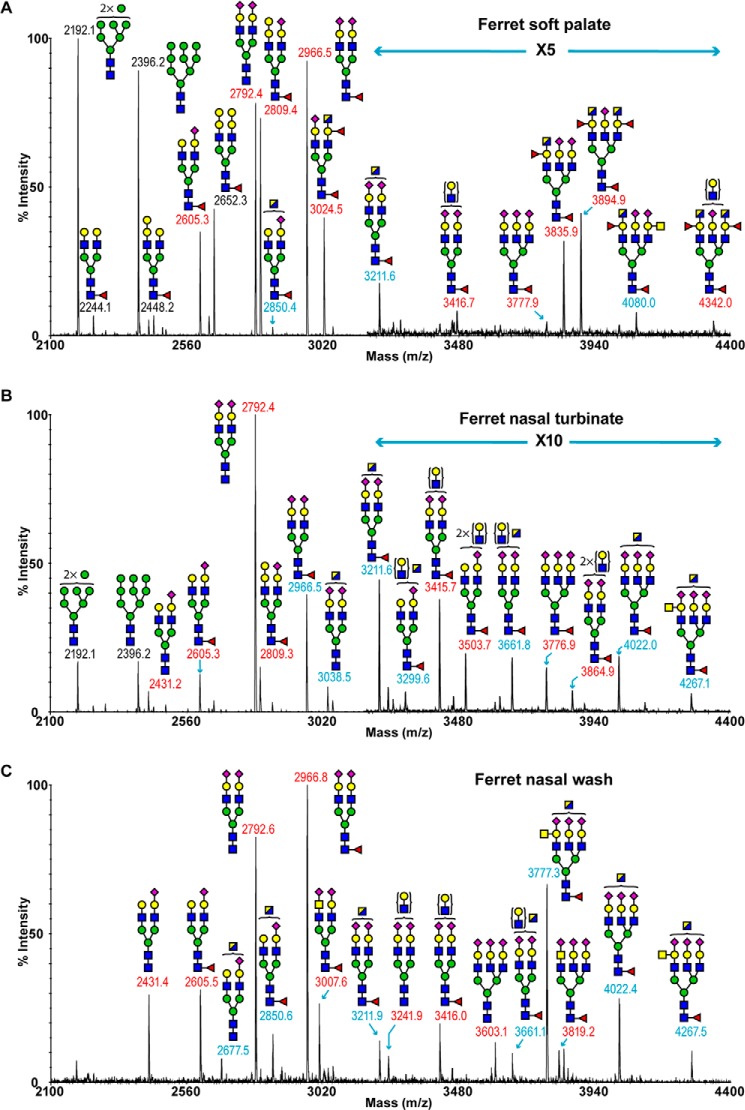
***N*-Glycan profiles of upper respiratory tract.** *A,* MALDI-TOF spectrum of permethylated *N*-glycans of ferret soft palate. *B,* MALDI-TOF spectrum of permethylated *N*-glycans of nasal turbinate. *C,* MALDI-TOF spectrum of permethylated *N*-glycans of nasal wash. Annotations include major structures that are unsialylated (*black*), sialylated (*red*), and glycans carrying the Sda epitope (*blue*). Putative structures are based on composition, tandem MS, and biosynthetic knowledge. See “Experimental Procedures” for full details of experiments that provided data for structural assignments. Structures that show sugars outside of a *bracket* have not been unequivocally defined. All molecular ions detected are present in the form [M + Na]^+^. Full lists of structural compositions are available in the supplemental Tables S8–10. Color symbols are as follows: *yellow square*, GalNAc; *blue square*, GlcNAc; *half-blue/half-yellow square*, GalNAc or GlcNAc; *yellow circle*, galactose; *green circle*, mannose; *purple diamond*, *N*-acetylneuraminic acid; *red triangle*, fucose.

The MALDI-TOF spectrum of ferret soft palate *N*-linked glycans is shown in Fig. 6*A* and supplemental Table S8. High mannose structures (*m*/*z* 2192 and 2396) were present as well as complex type sialo-glycans (*m*/*z* 2605–4342). Major nonreducing terminal capping groups were sialylated LacNAc (*m*/*z* 2605, 2792, 2966, 3211, and 3835), but the Sda epitope (*m/z* 2850, 3211, and 4080) and Galα1–3Gal capped species are also present (*m*/*z* 2448, 2652, and 2809). Also observed was a series of di- and tri-fucosylated structures whose compositions are consistent with the presence of the human blood group A epitope in some antennae, although we have not been able to unambiguously differentiate between a terminal GlcNAc/GalNAc (*m*/*z* 3024, 3835, 3894, 4080, and 4342). This and other structural features were confirmed by MS/MS analysis (supplemental Fig. S3). The ferret soft palate *O-*glycome is dominated by core 1 glycans (supplemental Fig. S4, *m*/*z* 534, 708, 895, 953, 1069, 1140, 1256, and 1501). Compositions consistent with Sda-containing species (*m*/*z* 1140, 1501, and 1589) and the potential human blood group A epitope (*m*/*z* 953) were also present.

The MALDI-TOF profiling of ferret nasal turbinate *N*-linked glycans indicated the presence of high mannose structures (Fig. 6*B* and supplemental Table S9), *m*/*z* 2192 and 2396, and a series of complex glycans with compositions that were consistent with core-fucosylated and bi- and tri-antennary structures (*e.g. m*/*z* 2966, 3211, 3661, 3776, and 4267) (Fig. 6*B*). Similar to the ferret lung, the major nonreducing end capping groups were NeuAc-Gal-GlcNAc (*e.g. m*/*z* 2792, 2966, 3415, 3503, and 3864) and the Sda epitope (*e.g. m*/*z* 3038, 3211, 3661, 4022, and 4267). Additionally, minor but significant levels of molecular ions with the Galα1–3Gal terminated structure were also observed (*m*/*z* 2809).

The MALDI-TOF spectrum of ferret nasal wash *N*-linked glycans was dominated by complex glycans with compositions corresponding to bi- and tri-antennary structures up to *m*/*z* 4400 (Fig. 6*C* and supplemental Table S10). Sialylated LacNAc (*e.g. m*/*z* 2431, 2792, 2966, 3241, 3777, and 4022) and the Sda epitope (*e.g.* 2850, 3211, 3661, and 4267) were the two most abundant nonreducing end-capping groups in addition to the expression of minor but significant levels of sialylated LacdiNAc structures (*m*/*z* 3007 and 3819). The ferret nasal wash *O-*glycome contains only core 1 glycans (supplemental Fig. S4, *m*/*z* 534, 895, 1140, and 1501). Compositions consistent with Sda-containing species (*m*/*z* 1140, 1501) were present.

##### Lectin Binding Histochemistry and Enzyme Immunohistochemistry

**FIGURE 7.**
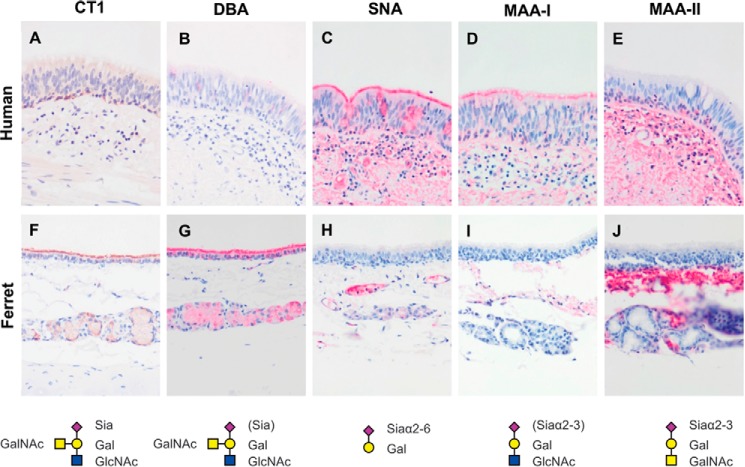
**Comparison of human and ferret lower airway tissues for sialylated glycans.** *A–E,* immunohistochemistry and lectin binding of human bronchial mucosa using the antibody CT1 (*A*) and the lectin DBA (*B*), SNA (*C*), MAA-I (*D*), and MAA-II (*E*), respectively. *F–J,* immunohistochemistry and lectin binding of ferret trachea using the antibody CT1 (*F*) and the lectin DBA (*G*), SNA (*H*), MAA-I (*I*), and MAA-II (*J*), respectively. Antibody CT1 identifies the Sda epitope; lectin DBA binds to terminal GalNAc; SNA binds to Siaα2–6-glycans; MAA-I binds to Siaα2–3-*N*-linked glycans, and MAA-II binds to Siaα2–3*-O-*linked glycans. Magnification ×200.

**FIGURE 8.**
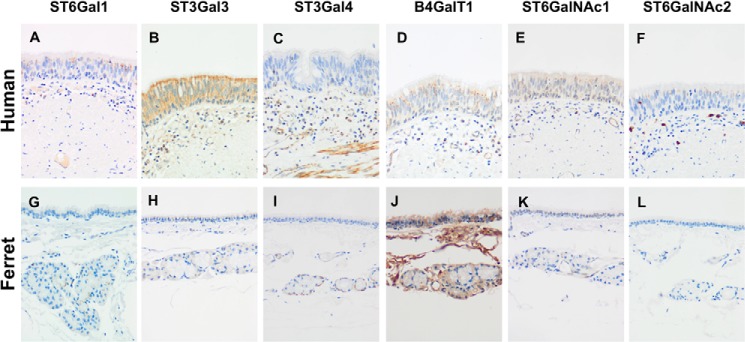
**Comparison of human and ferret lower airway tissues for sialyltransferases.** *A–F,* histochemistry of human bronchus identifying the sialyltransferases ST6Gal1 (*A*), ST3Gal3 (*B*), ST3Gal4 (*C*), β4GalT1 (*D*), ST6GalNAc1 (*E*), and ST6GalNAc2 (*F*), respectively. *G–L,* histochemistry of ferret trachea identifying the sialyltransferases ST6Gal1 (*G*), ST3Gal3 (*H*), ST3Gal4 (*I*), β4GalT1 (*J*), ST6GalNAc1(*K*), and ST6GalNAc2 (*L*), respectively. ST6Gal1, ST3Gal3, ST3Gal4, and β4GalT1 forms the β1–4Gal-GlcNAc bond; ST6GalNAc1 and ST6GalNAc2 are involved in Siaα2–6-*O-*glycan formation. Magnification ×100.

**FIGURE 9.**
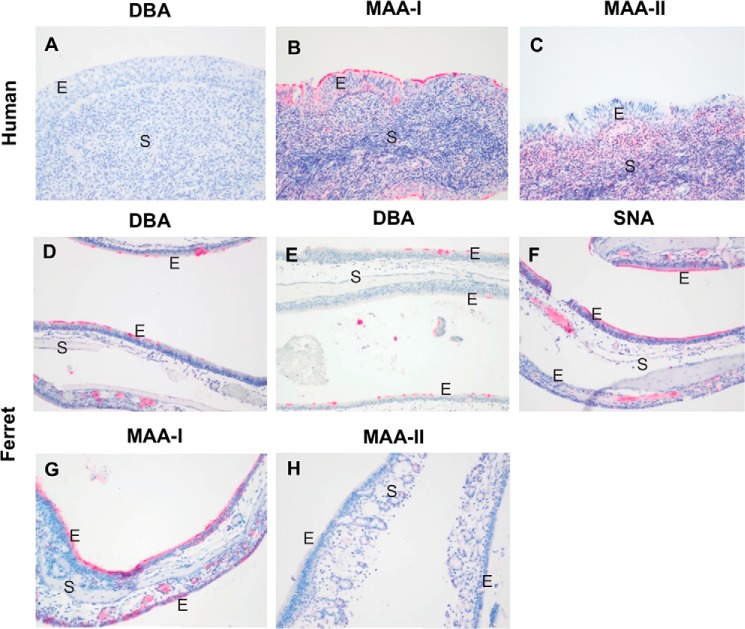
**Comparison of human and ferret upper airways.** *A,* lectin binding by DBA to the human nasopharynx. *B,* lectin binding by MAA-I to the human nasopharynx. *C,* lectin binding by MAA-II to the human nasopharynx. *D,* lectin binding of DBA to the ferret nasal mucosa. *E,* lectin binding by DBA to the ferret olfactory mucosa. *F,* lectin binding by SNA to the ferret nasal mucosa. *G,* lectin binding by MAA-I to the ferret nasal mucosa. *H,* lectin binding by MAA-II to the ferret nasal mucosa. *E,* epithelium; *S,* stroma. *A* and *B,* magnification ×200. *C–H,* magnification ×100.

**FIGURE 10.**
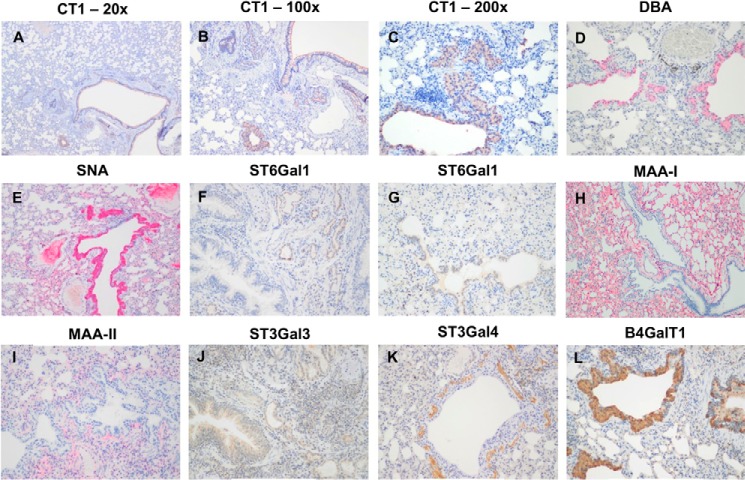
**Ferret lung staining for sialylated glycans and sialyltransferases.** *A–C,* ferret lung showing staining of the antibody CT1 in the magnification of ×20 (*A*), ×100 (*B*), and ×200 (*C*), respectively. *D,* ferret lung showing staining of the lectin DBA. *E,* ferret lung showing staining of the lectin SNA. *F* and *G,* ferret lung showing staining of the T6Gal1. *H,* ferret lung showing staining of the lectin MAA-I. *I,* ferret lung showing staining of the lectin MAA-II. *J–L,* ferret lung showing staining with ST3Gal3 (*J*), ST3Gal4 (*K*), and β4GalT1 (*L*), respectively. *D–L,* magnification ×200x.

To assess the expression of sialylated glycans between human and ferret tissues, lectin-binding studies were undertaken. The human bronchial mucosa (Fig. 7, *A–E*) showed strong binding with SNA and MAA-I with no apical or cytoplasmic binding by DBA or MAA-II identified. Stromal binding by MAA-II was noted. No cytoplasmic or apical staining for CT1 was seen though the basal epithelium. In contrast, the ferret tracheal mucosa (Fig. 7, *F–J*) showed strong binding by DBA to the apical surface of the ciliated cells and also to the cytoplasm of the submucosal glands. SNA binding was patchy, and there was no apical binding with MAA-I or MAA-II, although stromal binding with MAA-II was noted. Punctate staining corresponding to Golgi regions was seen in the human bronchial mucosa with antibodies against ST6Gal1, β4GalT1, and ST6GalNAc1 (Fig. 8) with more intense cytoplasmic staining with ST3Gal3. ST3Gal4 staining was localized to myoepithelial cells. In the ferret tracheal epithelium and glands, there was also punctate staining seen with ST6Gal1 (supplemental Fig. S5, *A* and *B*) but no cytoplasmic staining of the epithelium was seen with other enzymes apart from β4GalT1. Immunohistochemistry for β4GalNT2, the enzyme that attaches a terminal GalNAc to form the Sda epitope, showed expression in the cytoplasm of the ferret tracheal epithelium and glands (supplemental Fig. S5*C*). As a positive control, we used mouse kidney showing staining of the tubules where the Tamm-Horsfall glycoprotein is synthesized (supplemental Fig. S5*D*). This glycoprotein has been previously shown to be extensively sialylated with Sda. There was no staining of human nasopharyngeal mucosa with DBA or MAA-II (Fig. 9, *A* and *C*), and staining of the ferret nasal mucosa showed a heterogeneous binding by SNA, DBA, and MAA-I with no significant binding of MAA-II. (Fig. 9, *D–H*). There was no difference in binding of DBA to Asian and Caucasian lung tissues (supplemental Fig. S6). The ferret lung (Fig. 10) showed a similar pattern to the trachea in that most of the binding of lectins was confined to the terminal bronchioles with little binding to alveoli apart from MAA-I. In contrast to the trachea, there was more staining with ST3Gal3 noted.

##### Glycan Array

**FIGURE 11.**
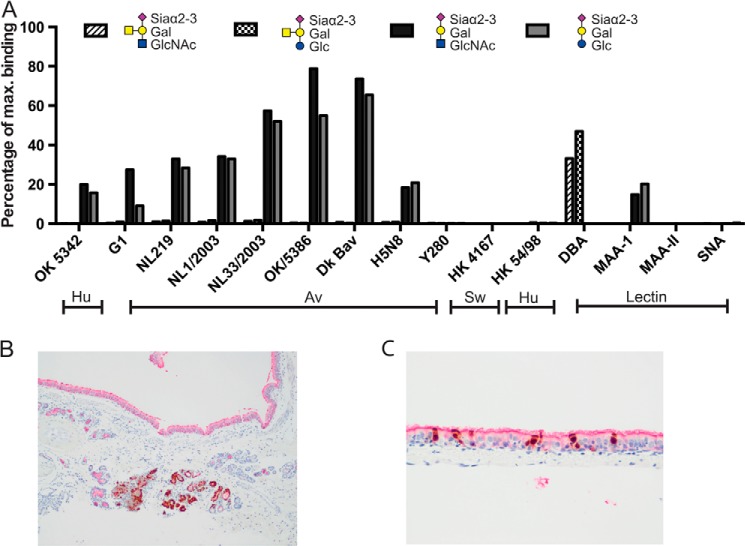
**Glycan array for Sda epitope and virus immunohistochemistry.** *A,* glycan array analysis of binding of avian (*Av*), human (*Hu*), and swine (*Sw*) viruses and the lectins DBA, SNA, MAA-I, and MAA-II to GalNAcβ1–4(Neu5Acα2–3)Galβ1–4GlcNAcβ-Sp8, GalNAcβ1–4(Neu5Acα2–3)Galβ1–4Glcβ-Sp0, Neu5Acα2–3Galβ1–4GlcNAcβ-Sp8, and Neu5Acα2–3Galβ1–4Glcβ-Sp0. *B,* double staining immunohistochemistry of ferret bronchus for the lectin DBA (*red color*) and influenza nucleoprotein (*brown color*) of ferrets infected with H1N1pdm. *C,* double staining immunohistochemistry of ferret trachea for the lectin DBA (*red color*) and influenza nucleoprotein (*brown color*) of ferrets infected with H1N1pdm. *B,* magnification ×100. *C,* magnification ×200.

Assessment of glycan array binding data showed that, as expected, avian viruses of the H7N7, H5N8, and H1N1 subtype bound glycans with a terminal Siaα2-3 configuration although human H3N2 and H1N1 viruses and H9N2 Y280 subtype did not (Fig. 11*A*). Notably the addition of the GalNAc side chain to produce the Sda epitope completely abolished binding by the human and avian viruses. The lectin DBA recognized the Sda epitope but MAA-I or MAA-II did not. As expected, SNA did not bind to any of the Siaα2–3-terminated glycans. There was co-localization of influenza nucleoprotein with DBA indicating that the presence of Sda was not a barrier to infection with α2–6-binding viruses (Fig. 11*B*).

## DISCUSSION

Our integrated glycomic strategies coupled with lectin staining and immunohistochemistry experiments have shown that there is a heterogeneous expression of α2–3- and α2–6-linked sialic acids with the latter being more abundant. The ferret respiratory tract displays a diverse range of *N*-linked glycans. The major structures were sialylated complex type multiantennary glycans, many of which carry LacNAc extensions. Three classes of sialylated structures were identified, namely sialylated LacNAc, the Sda epitope, and sialylated LacdiNAc, with the first two being the most abundant. The prominent display of the Sda structure was also detected in both *O-*linked and glycolipid-derived glycans. Other minor terminal modifications observed included the blood group A epitope and the Galα1–3Gal motif.

The respiratory tract of ferret displays a tissue-specific glycosylation as we observed a differential expression of structures in different tissues. From the lower to upper airway, there is a reduced expression of multiantennary complex glycans as well as other high *m*/*z* structures in the soft palate and nasal turbinate in comparison with the lung and trachea. However, it should be noted that a more limited amount of tissue for glycomic analysis was available, which could inhibit the detection of higher molecular weight glycan species. The trachea and soft palate showed the highest expression of the Galα1–3Gal motif. Of particular note is the display of potential blood group A epitopes that are carried by bi- and tri-antennary glycans of significant levels in soft palate but only in minute quantities on other tissues. The blood group A epitope has been reported previously by characterization of ferret respiratory tract mucins (33), but curiously, the ferret does not appear to express blood group epitopes on red blood cells (34).

We have previously characterized the glycomic profiles of respiratory tract tissues from human, swine, and mouse, which allow a comparison of potential influenza receptors to be made (19–21). Data generated from this study for the first time enable us to validate the suitability of the ferret model over other alternative animals that are utilized to investigate influenza host infectivity. All the respiratory tissues analyzed to date express both α2–3- and α2–6-linked Sia, with the human expressing the highest proportion of α2–6-linked Sia and the mouse expressing the highest proportion of α2–3-linked Sia. However, two terminal structures, NeuGc and Galα1–3Gal, are not present in human (23, 24) but are expressed abundantly by both swine and mouse. In contrast, ferret respiratory tissues only contain a small amount of Galα1–3Gal-terminated glycans, and the presence of NeuGc is below the limit of detection. From this point of view, the respiratory tract of the ferret shows a much greater degree of similarity to the human airway than swine and mouse. Nonetheless, the expression of Sda and sialylated LacdiNAc in ferret appears to be unique, because these structures were not detected in the respiratory tissues of human, mouse, or swine by mass spectrometry. Thus, to further validate the ferret model, understanding the structural and functional properties of these epitopes is crucial.

Although interaction studies with glycan arrays, which contain Sda, have been previously reported, the potential significance of interactions between the Sda tetrasaccharide and influenza hemagglutinin has been largely ignored. As expected, human influenza virus, including a series of pandemic H1N1 strains, did not show any interaction with the Sda epitope (35, 36). This is because the human viruses have preferential binding toward α2–6-linked sialic acids, whereas the Sda carries α2–3-NeuAc. Surprisingly, avian strains such as H5N1 failed to recognize the Sda epitope even though they mainly bind α2–3-sialosides (37, 38). In addition, we demonstrated that the Sda is resistant to digestion with neuraminidase with α2–3 specificity (Figs. 2–4). The resistant characteristic of the Sda is likely due to the close proximity between the GalNAc and NeuAc. It has been proposed that substitution of the 4-hydroxy group of the galactose with a GalNAc residue shields the nearby sialic acid molecule from the binding site of external enzymes, as the removal of the GalNAc significantly improves the susceptibility of Sda to neuraminidase treatment (30, 39). Therefore, the sialic acid residue may not be readily accessible to the α2,3-specific hemagglutinin, and therefore the Sda-carrying structures that are present along the respiratory tract of ferret could potentially serve as a protective mechanism against infection with avian strains of influenza virus by diluting the density of authentic Sia receptors.

The LacdiNAc motif is not only displayed by glycoproteins of some lower animal species but is also expressed by mammalian glycoproteins such as lutropin and glycodelin (40, 41). Previous glycan array studies indicate the α2–6-sialylated LacdiNAc can be recognized by the seasonal H1N1 influenza virus and the droplet-transmissible H5N1 strain (42, 43). Therefore, the sialylated LacdiNAc structure (NeuAcα2–6GalNAcβ1-4GlcNAc) is also a potential viral receptor to the influenza virus. However, this trisaccharide motif has not yet been detected in human respiratory tissues.

The Sda epitope was also detected by lectin binding studies using DBA and immunohistochemistry using an antibody against β4GalNT2, which adds the GalNAc side chain. In contrast to the ferret respiratory tracts, we found no evidence of DBA binding to the human nasopharynx respiratory or pulmonary epithelium. In the latter case, we used Asian and Caucasian tissues as in humans the Sda antigen is inherited as an autosomal dominant character with racial differentiation, where it is present in tissues and secretions, in addition to red cell membranes, where it is a component of the rare Cad phenotype. Even though previous studies suggested that the Sda had limited expression in human lung samples, our lectin binding studies and immunohistochemistry showed that in ∼70% of bronchial samples there is a low level of DBA and CT1 staining to occasional submucous glands.

Our MS and lectin binding studies may help to explain why the ferret trachea is a less favored site for α2–3-binding avian virus replication compared with the nasal turbinates. Our findings indicate this decreased replication ability may be attributed to the overexpression of Sda and Galα1–3Gal. The addition of the GalNAc side chain to glycoproteins and glycolipids to form this Sda epitope significantly inhibits binding to α2–3-binding viruses on a glycan array. It should be noted that the Sda epitope also restricted binding by MAA-I and MAA-II to α2–3-sialylated glycans. Also, the cleavage ability of this epitope by viral neuraminidases has not been investigated since the 1960s (31), but previous publications have shown a reduced cleavage by certain bacterial sialidases (29).

The efficient replication of influenza viruses in the ferret nasal turbinates was first explored by Francis and Stuart-Harris (44) who demonstrated two different types of epithelium, a standard respiratory-type epithelium and a modified olfactory-type epithelium. There have been limited studies on the distribution of α2–3- *versus* α2–6-sialylated receptors in this area. Roberts *et al.* (45) showed that there was strong binding of SNA to the epithelium but little binding of MAA (which contains both isoforms of MAA). We found that the binding of SNA and MAA-I was heterogeneous and that there was also heterogeneous binding of DBA to the ferret nasal epithelium; however, similar to the human nasopharynx, we found little evidence of binding of MAA-II, which identifies *O-*linked glycans, but it should be remembered that the formation of the Sda epitope restricted binding by MAA-II to α2–3 *O-*glycans. In contrast to the ferret upper respiratory tract, we found no evidence of DBA binding to the epithelium of the human nasopharynx.

In conclusion our mass spectrometric, lectin binding, and immunohistochemical findings indicate that the ferret respiratory tract tissues differ from human respiratory tract tissues in the expression of sialylated glycans. In particular, we have found that ferret samples contain the Sda epitope. We hypothesize that expression of this epitope competes with expression of sialylated structures that are recognized by the virus and therefore could reduce binding by α2–3-Sia binding influenza viruses. Other differences in the ferret respiratory tract tissues compared with the human include the expression of the sialylated LacdiNAc and Galα1–3Gal structures, which have not been identified in the human respiratory tract. The significance of these other structures in respect to the extrapolation of influenza transmission experiments in ferret studies to the human situation needs further exploration.

## Supplementary Material

Supplemental Data

## Footnotes

4 The abbreviations used are:

Sia: sialic acid
NeuAc: *N*-acetylneuraminic acid
NeuGc: *N*-glycolylneuraminic acid
LacNAc: *N*-acetyllactosamine
LacdiNAc: *N,N*′-diacetyllactosamine
MAA: *Maackia amurensis* agglutinin
SNA: *Sambucus nigra* agglutinin
DBA: *Dolichos biflorus* agglutinin
rEGCase II: recombinant endoglycoceramidase II.
