# Supplementary material for: Glycomic Characterization of Respiratory Tract Tissues of Ferrets: IMPLICATIONS FOR ITS USE IN INFLUENZA VIRUS INFECTION STUDIES
Source: J Biol Chem. 2014 Aug 18;289(41):28489–504. doi: 10.1074/jbc.M114.588541 (PMC4192499; doi:10.1074/jbc.M114.588541)
Supplement: Supplemental Data [file supp_289_41_28489__index.html]

Glycomic characterisation of respiratory tract tissues of ferrets: implications for its use in influenza virus infection studies — Glycomic Characterization of Respiratory Tract Tissues of Ferrets — Glycomic Profiles of Ferret Respiratory Tissues — Supplemental Data 

# Glycomic Characterization of Respiratory Tract Tissues of Ferrets

## Supplemental Data

**Files in this Data Supplement:**

- supplementary Figures S1-6 (.docx, 6.7 MB) - supplementary Figures S1-6
- supplementary Tables S1-10 (.xls, 60 KB) - supplementary Tables S1-10
