# Supplementary material for: Glycomic Characterization of Respiratory Tract Tissues of Ferrets: IMPLICATIONS FOR ITS USE IN INFLUENZA VIRUS INFECTION STUDIES
Source: J Biol Chem. 2014 Aug 18;289(41):28489–504. doi: 10.1074/jbc.M114.588541 (PMC4192499; doi:10.1074/jbc.M114.588541)
Supplement: Supplemental Data [file supp_M114.588541_jbc.M114.588541-1.docx]

Glycomic Characterisation of Respiratory Tract Tissues of Ferrets: Implications for its Use in Influenza Virus Infection Studies.

**Nan Jia, Wendy S. Barclay, Kim Roberts, Hui-Ling Yen, Renee W.Y. Chan, Alfred K.Y. Lam, Gillian Air, JS. Malik Peiris, Anne Dell, John M. Nicholls and Stuart M. Haslam**

**SUPPLEMENTARY DATA**

Figure Legends:

**Supplementary Figure S1.** **MS/MS analysis of Sda presenting structures.** The MALDI-TOF/TOF fragmentation of permethylated N-glycan expressing the Sda epitope at m/z 3252 which was derived from the ferret lung. Detailed analysis of the spectra revealed the presence of two potential structural isomers which for greater clarity are annotated in separate panels. A. The fragmentation of molecular ion at m/z 3252 produced a B-ion at 1092 represented the tetrasaccharide constituent of Sda capping group whist its corresponding Y-ion at 2183 indicated the loss of Sda epitope from the molecular ion. The B-ion of m/z 888 was the signature ion for sialyalted LacdiNAc unit together with its complementary Y-ion at 2387. Taken together these data are consistent with a bi-antennary core fucosylated complex structure which has one Sda antenna and one sialyalted LacdiNAc antenna. B. The fragmentation of molecular ion at m/z 3252 also produced a B-ion at 847 with its corresponding Y-ion at 2428 indicated a sialyalted LacNAc antenna. The B-ion of m/z 888 was the signature ion for sialyalted LacdiNAc unit together with its complementary Y-ion at 2387. Taken together these data are consistent with a bi-antennary core fucosylated complex structure which has one sialyalted LacNAc antenna and one sialyalted LacdiNAc antenna. C. The MALDI-TOF/TOF spectrum of permethylated O-glycan expressing the Sda epitope at m/z 1501 which was derived from the ferret lung. D. The MALDI-TOF/TOF spectrum of permethylated, deuteroreduced glycolipid expressing the Sda epitope at m/z 1998 which was derived from the ferret lung. (Color symbols: yellow square, GalNAc; blue square, GlcNAc; half-blue/half-yellow square, GalNAc or GlcNAc; yellow circle, galactose; green circle, mannose; purple diamond, N-acetylneuraminic acid; red triangle, fucose).

**Supplementary Figure S2.** **Partial N-glycan profile of ferret lung after the treatment with Endo-β-Galactosidase.** The partial MALDI-TOF spectrum of permethylated N-glycans was derived from the ferret lung, after digestion with the Endo-β-Galactosidase. The molecular ions at m/z 518 and 722 indicated the presence of poly-LacNAc extensions. Non-reducing end capping groups were also observed including the Galα-Gal motif (m/z at 926), sialylated LacNAc (m/z 1083) and Sda epitope (m/z 879 and 1328). Annotations include major structures that are un-sialylated (black), sialylated (red) and glycans carrying the Sda epitope (blue).All molecular ions detected are present in the form [M+Na]^+^. (Color symbols: yellow square, GalNAc; blue square, GlcNAc; half-blue/half-yellow square, GalNAc or GlcNAc; yellow circle, galactose; green circle, mannose; purple diamond, N-acetylneuraminic acid; red triangle, fucose).

**Supplementary Figure S3.** **MS/MS analysis of blood group A presenting structure.** The MALDI-TOF/TOF spectrum of the permetylated N-glycan was derived from the ferret soft palate. The B-ion of m/z 905 was the signature ion for the blood group A tetrasaccharide together with its complementary Y- ion at 2142. (Color symbols: yellow square, GalNAc; blue square, GlcNAc; half-blue/half-yellow square, GalNAc or GlcNAc; yellow circle, galactose; green circle, mannose; purple diamond, N-acetylneuraminic acid; red triangle, fucose).

**Supplementary Figure S4.** **O-glycan profiles of soft palate and nasal wash.** A. The MALDI-TOF spectrum of permethylated O-glycans that were derived from ferret soft palate. B. The MALDI-TOF spectrum of permethylated O-glycans that were derived from ferret nasal wash. Annotations include major structures that are un-sialylated (black), sialylated (red) and glycans carrying the Sda epitope (blue). All molecular ions detected are present in the form [M+Na]^+^. Putative structures are based on composition, tandem MS, and biosynthetic knowledge. Structures that show sugars outside of a bracket have not been unequivocally defined. (Color symbols: yellow square, GalNAc; blue square, GlcNAc; half-blue/half-yellow square, GalNAc or GlcNAc; yellow circle, galactose; purple diamond, N-acetylneuraminic acid; red triangle, fucose).

**Supplementary Figure S5. ST6Gal1 and ß4GalNT2 immunohistochemistry.** A. Immunohistochemistry for ST6Gal1 in ferret tracheal epithelium. B. Immunohistochemistry for ST6Gal1 in ferret tracheal glands. C. Immunohistochemistry for β4GalNT2 in ferret trachea. D. Immunohistochemistry for β4GalNT2 in mouse kidney. A and B: Magnification 400x. C and D: Magnification 200x.

**Supplementary Figure S6. Comparison of Asian and Caucasian lungs for DBA binding.** Lectin binding of DBA to human lung tissues obtained from Asian and Caucasian lobectomy samples. Magnification x 40.

**Supplementary Table S1.** Compositional assignments, observed mass to charge ratio and relative abundance of singly charged sodiated molecular ions, [M+Na]+, observed in MALDI-TOF spectra of permethylated N-glycans derived from ferret lung. The peak list and assignment was generated and assigned manually based on 12C isotopic composition together with knowledge of the biosynthetic pathways. Relative abundance are presented as % calculated by dividing the signal intensity at the indicated mass by the signal intensity for the most abundant assigned glycan.

**Supplementary Table S2.** GC-MS analyses of partially methylated alditol acetates obtained from the 50% acetonitrile fraction of PNGase F released N-glycans of ferret lung.

**Supplementary Table S3.** Compositional assignments, observed mass to charge ratio and relative abundance of singly charged sodiated molecular ions, [M+Na]^+^, observed in MALDI-TOF spectra of permethylated O-glycans derived from ferret lung. The peak list and assignment was generated and assigned manually based on 12C isotopic composition together with knowledge of the biosynthetic pathways. Relative abundance are presented as % calculated by dividing the signal intensity at the indicated mass by the signal intensity for the most abundant assigned glycan.

**Supplementary Table S4.** Compositional assignments, observed mass to charge ratio and relative abundance of singly charged sodiated molecular ions, [M+Na]^+^, observed in MALDI-TOF spectra of permethylated, deuteroreduced glycolipids derived from ferret lung. The peak list and assignment was generated and assigned manually based on 12C isotopic composition together with knowledge of the biosynthetic pathways. Relative abundance are presented as % calculated by dividing the signal intensity at the indicated mass by the signal intensity for the most abundant assigned glycan.

**Supplementary Table S5.** Compositional assignments, observed mass to charge ratio and relative abundance of singly charged sodiated molecular ions, [M+Na]^+^, observed in MALDI-TOF spectra of permethylated N-glycans derived from ferret trachea. The peak list and assignment was generated and assigned manually based on 12C isotopic composition together with knowledge of the biosynthetic pathways. Relative abundance are presented as % calculated by dividing the signal intensity at the indicated mass by the signal intensity for the most abundant assigned glycan.

**Supplementary Table S6.** Compositional assignments, observed mass to charge ratio and relative abundance of singly charged sodiated molecular ions, [M+Na]^+^, observed in MALDI-TOF spectra of permethylated O-glycans derived from ferret trachea. The peak list and assignment was generated and assigned manually based on 12C isotopic composition together with knowledge of the biosynthetic pathways. Relative abundance are presented as % calculated by dividing the signal intensity at the indicated mass by the signal intensity for the most abundant assigned glycan.

**Supplementary Table S7.** Compositional assignments, observed mass to charge ratio and relative abundance of singly charged sodiated molecular ions, [M+Na]^+^, observed in MALDI-TOF spectra of permethylated, deuteroreduced glycolipids derived from ferret trachea. The peak list and assignment was generated and assigned manually based on 12C isotopic composition together with knowledge of the biosynthetic pathways. Relative abundance are presented as % calculated by dividing the signal intensity at the indicated mass by the signal intensity for the most abundant assigned glycan.

**Supplementary Table S8.** Compositional assignments, observed mass to charge ratio and relative abundance of singly charged sodiated molecular ions, [M+Na]^+^, observed in MALDI-TOF spectra of permethylated N-glycans derived from ferret soft palate. The peak list and assignment was generated and assigned manually based on 12C isotopic composition together with knowledge of the biosynthetic pathways. Relative abundance are presented as % calculated by dividing the signal intensity at the indicated mass by the signal intensity for the most abundant assigned glycan.

**Supplementary Table S9.** Compositional assignments, observed mass to charge ratio and relative abundance of singly charged sodiated molecular ions, [M+Na]^+^, observed in MALDI-TOF spectra of permethylated N-glycans derived from ferret nasal turbinate. The peak list and assignment was generated and assigned manually based on 12C isotopic composition together with knowledge of the biosynthetic pathways. Relative abundance are presented as % calculated by dividing the signal intensity at the indicated mass by the signal intensity for the most abundant assigned glycan.

**Supplementary Table S10.** Compositional assignments, observed mass to charge ratio and relative abundance of singly charged sodiated molecular ions, [M+Na]+, observed in MALDI-TOF spectra of permethylated N-glycans derived from ferret nasal wash. The peak list and assignment was generated and assigned manually based on 12C isotopic composition together with knowledge of the biosynthetic pathways. Relative abundance are presented as % calculated by dividing the signal intensity at the indicated mass by the signal intensity for the most abundant assigned glycan.

**Figure S1**


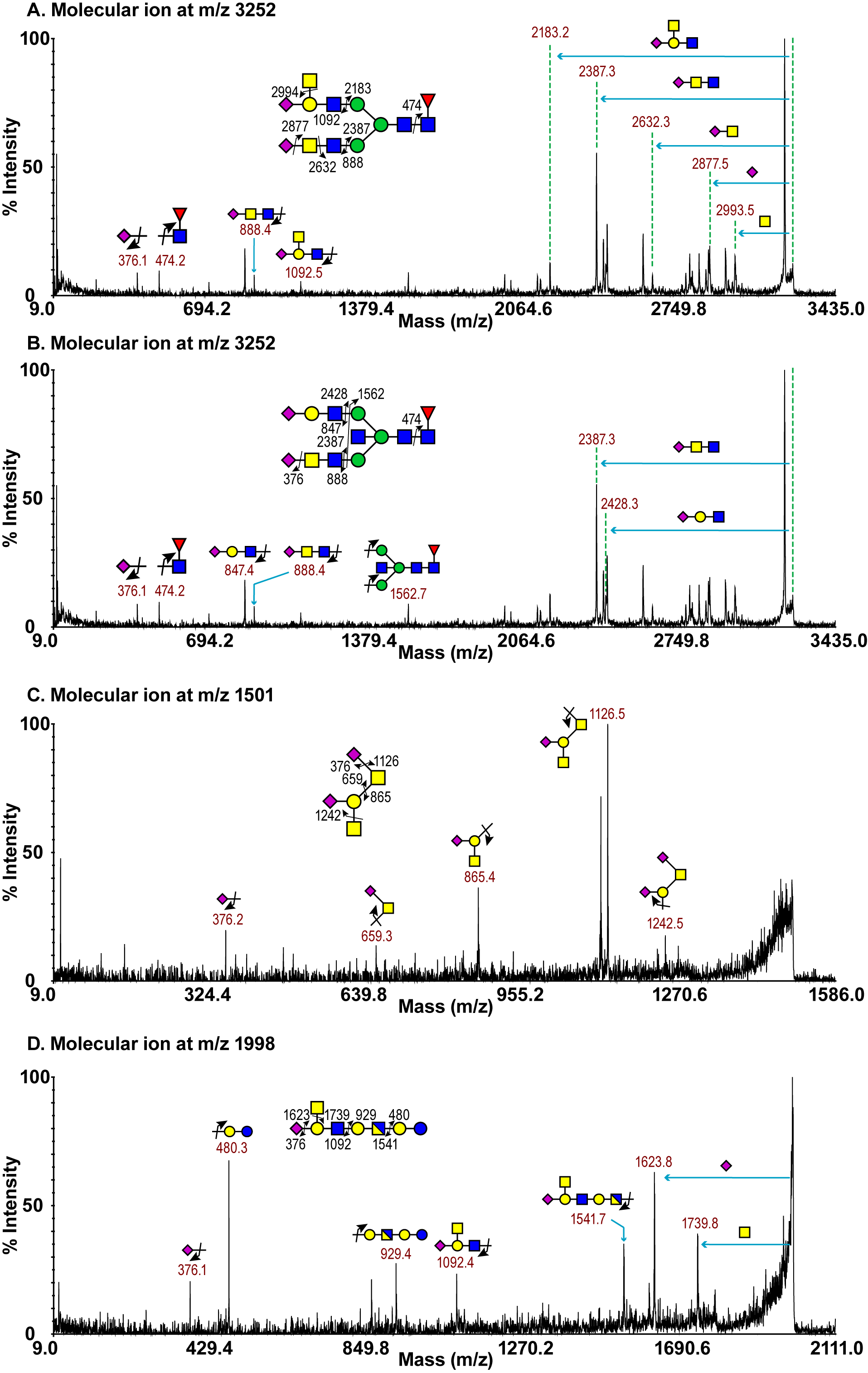


**Figure S2**


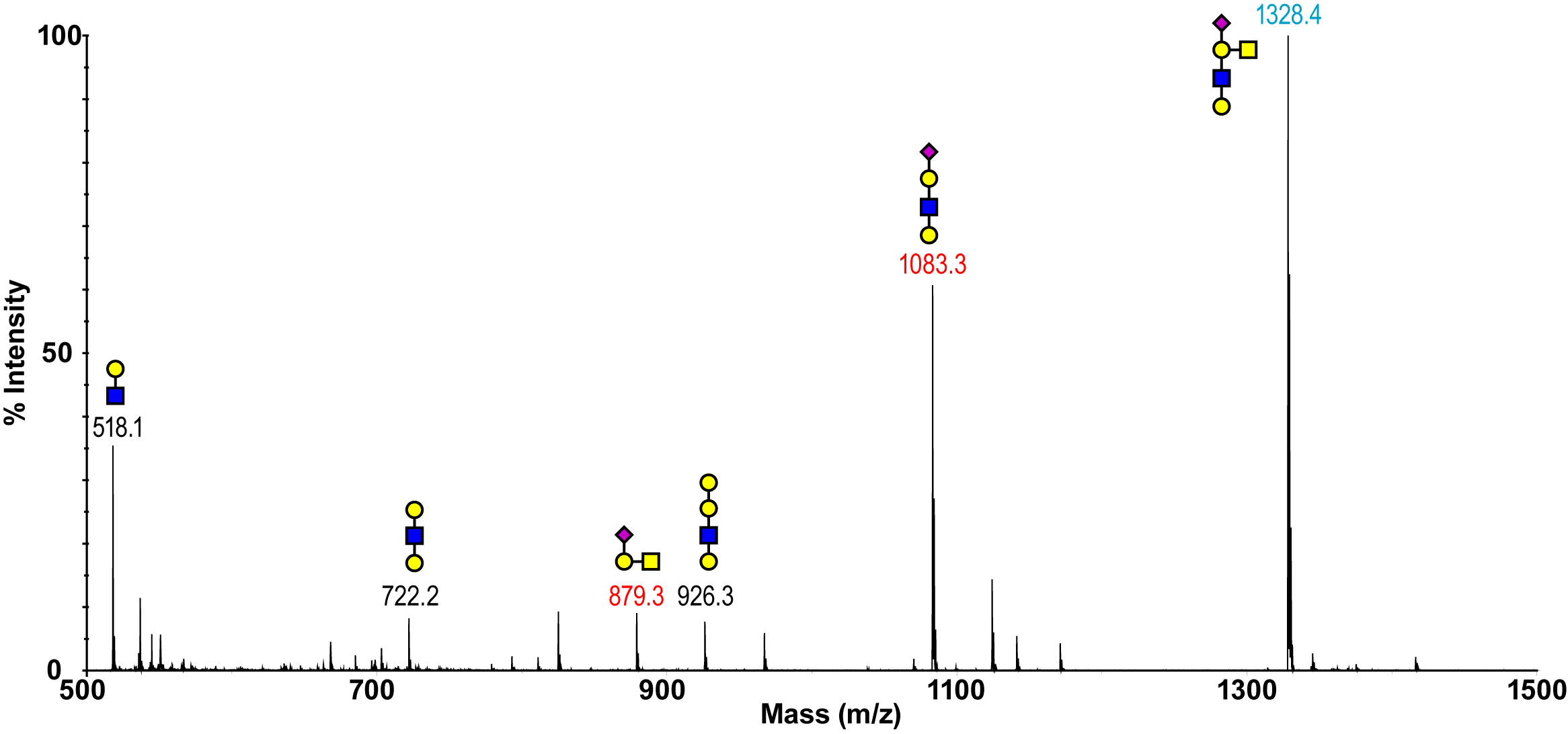


**Figure S3**


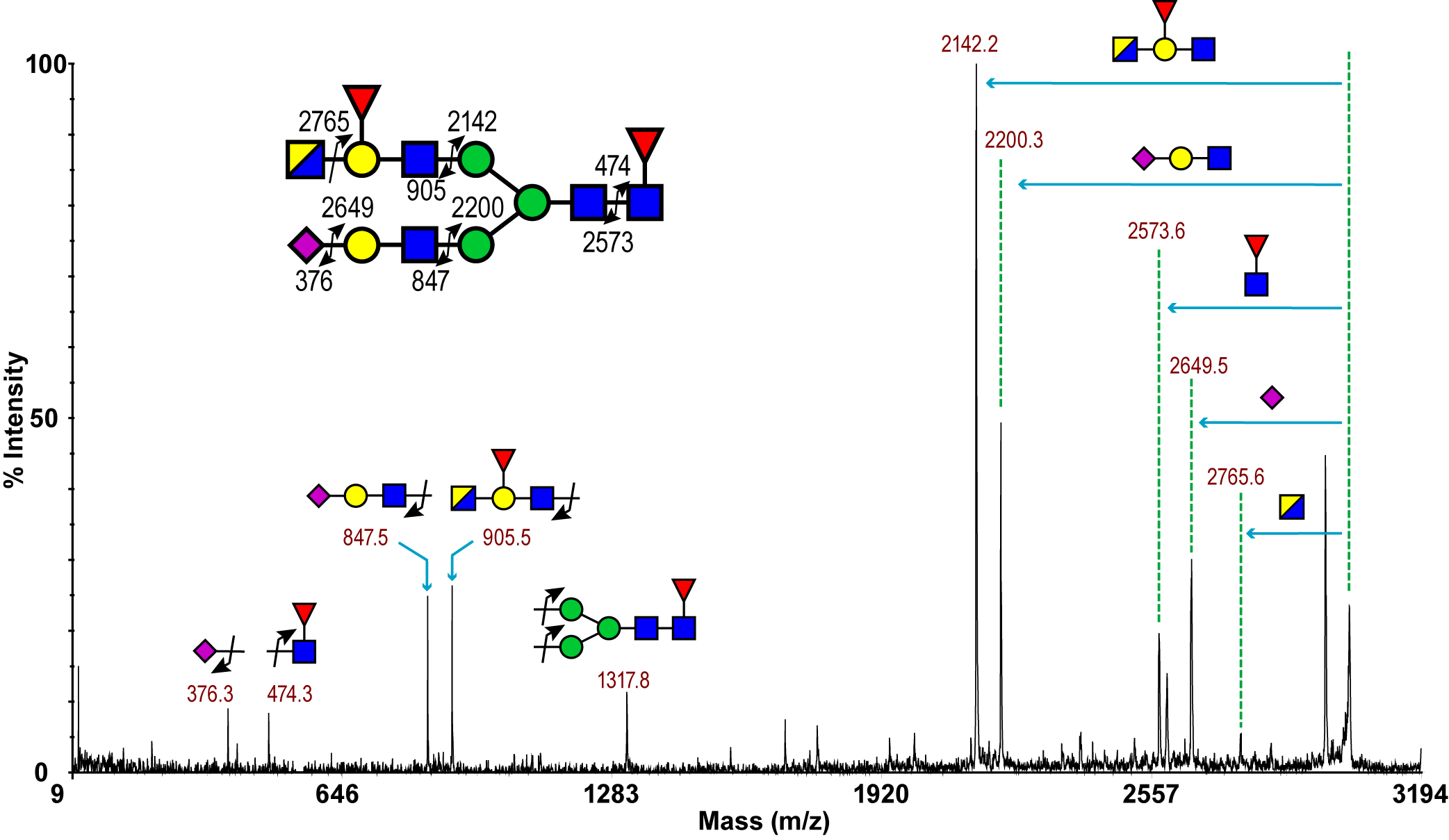


**Figure S4**


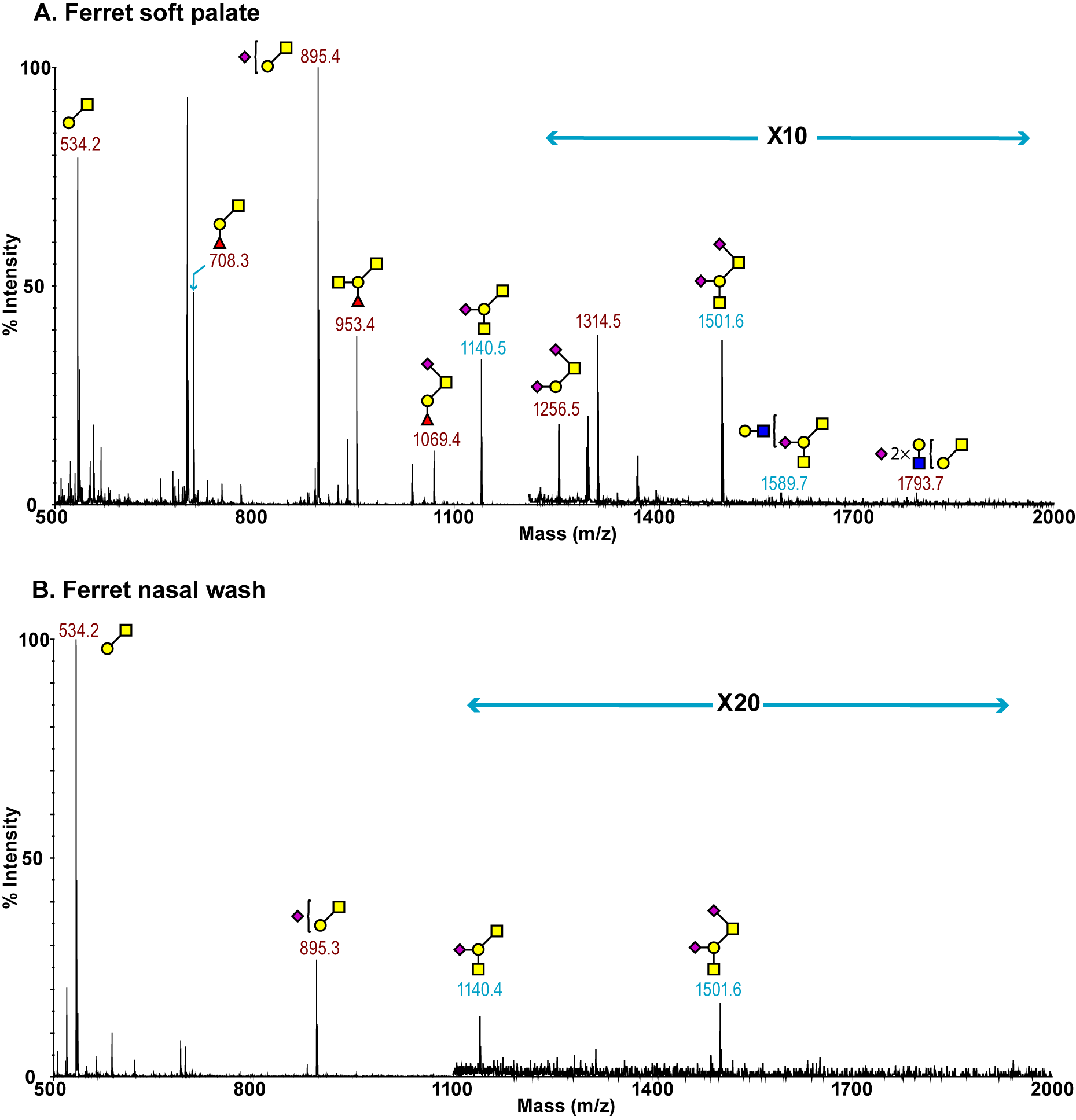


**Figure S5**


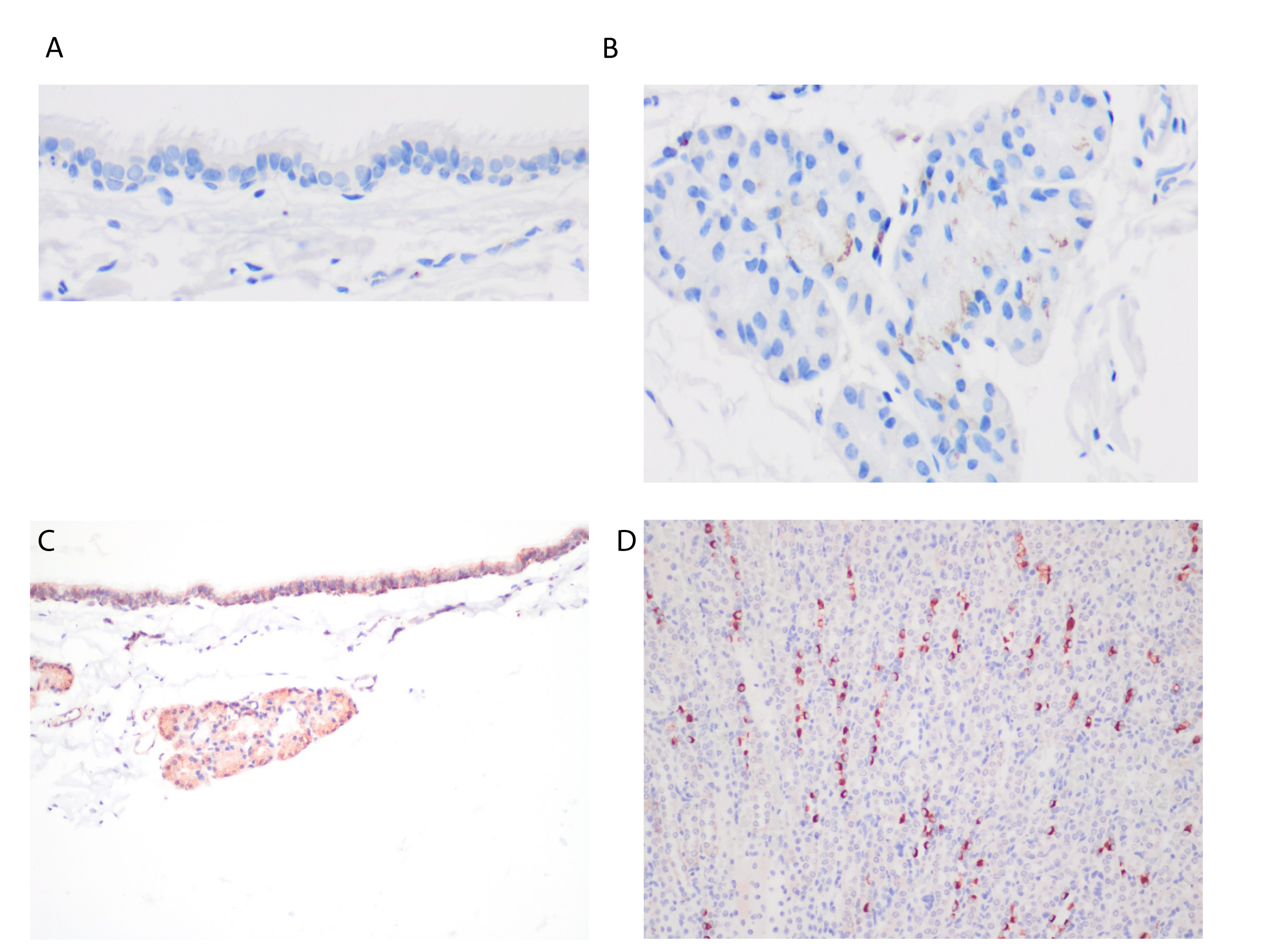


**Figure S6**

**
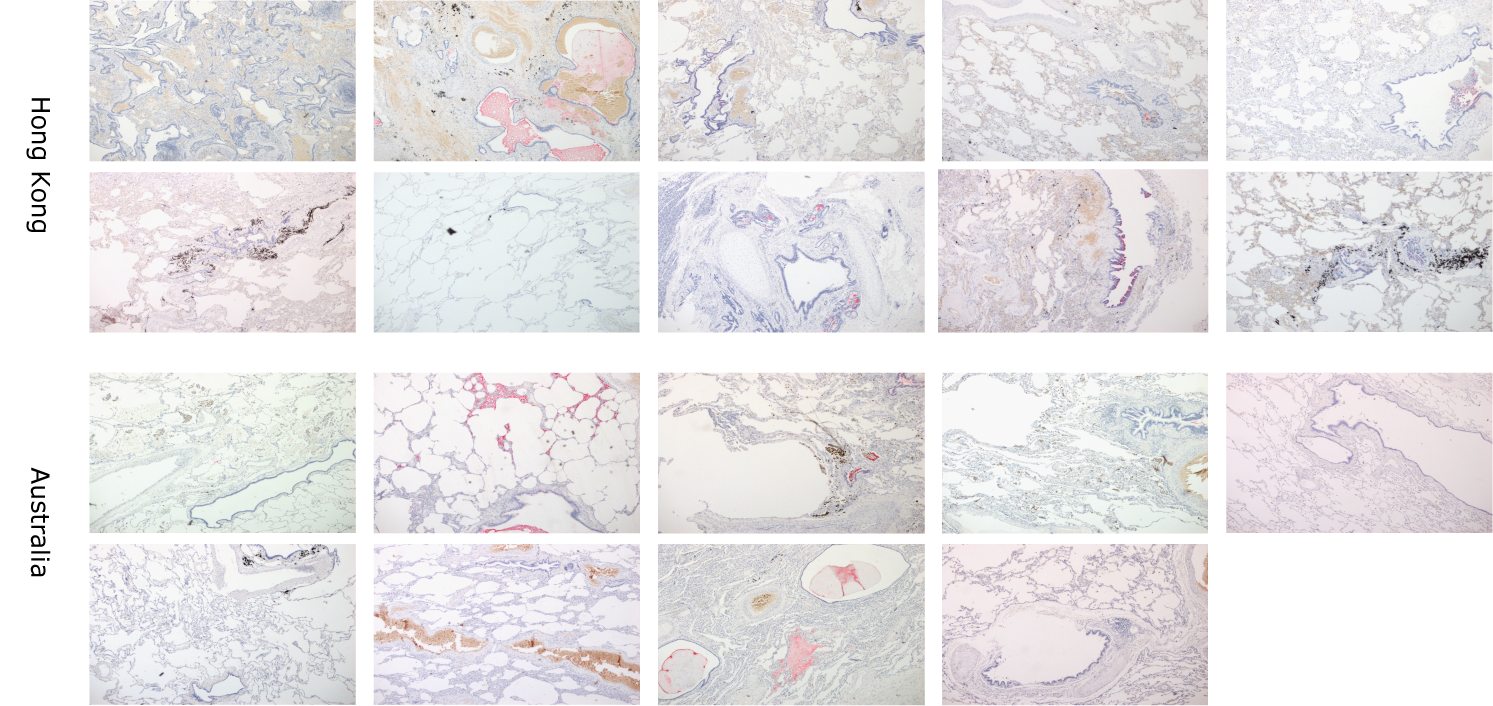
**
